# Predicting plant trait dynamics from genetic markers

**DOI:** 10.1038/s41477-025-01986-y

**Published:** 2025-04-17

**Authors:** David Hobby, Hao Tong, Marc Heuermann, Alain J. Mbebi, Roosa A. E. Laitinen, Matteo Dell’Acqua, Thomas Altmann, Zoran Nikoloski

**Affiliations:** 1 Systems Biology and Mathematical Modeling, Max Planck Institute of Molecular Plant Physiology, Potsdam, Germany; 2 Bioinformatics Department, Institute of Biochemistry and Biology, University of Potsdam, Potsdam, Germany; 3 Department of Molecular Genetics, Leibniz Institute of Plant Genetics and Crop Plant Research, Seeland OT Gatersleben, Gatersleben, Germany; 4 Organismal and Evolutionary Biology Research Programme, Viikki Plant Science Centre, University of Helsinki, Helsinki, Finland; 5 Institute of Plant Sciences, Scuola Superiore Sant’Anna, Pisa, Italy

**Keywords:** Plant development, High-throughput screening, Quantitative trait

## Abstract

Molecular and physiological changes across crop developmental stages shape the plant phenome and render its prediction from genetic markers challenging. Here we present dynamicGP, an efficient computational approach that combines genomic prediction with dynamic mode decomposition to characterize the temporal changes and to predict genotype-specific dynamics for multiple morphometric, geometric and colourimetric traits scored by high-throughput phenotyping. Using genetic markers and data from high-throughput phenotyping of a maize multiparent advanced generation inter-cross population and an *Arabidopsis thaliana* diversity panel, we show that dynamicGP outperforms a baseline genomic prediction approach for the multiple traits. We demonstrate that the developmental dynamics of traits whose heritability varies less over time can be predicted with higher accuracy. The approach paves the way for interrogating and integrating the dynamical interactions between genotype and environment over plant development to improve the prediction accuracy of agronomically relevant traits.

## Abstract (web summary)

This study presents a computational approach that effectively uses time-resolved multi-trait data from a high-throughput phenotyping platform for the genomic prediction of morphometric, geometric and colourimetric traits of *Arabidopsis* and maize lines.

## Main

The phenome of a plant comprises the entirety of traits expressed at any given time and is the integrated outcome of the effects of genetic factors, environmental conditions and their complex interactions. Understanding how the crop phenome changes over time can help predict individual traits at specific timepoints in crop development. However, this problem is challenging not only because of the intricate dependence between individual traits but also because of differences in how the phenomes of specific genotypes change over the plant life cycle.

The classical approach of genomic prediction (GP) in crops trains machine learning models using data on traits measured in a population of genotypes at a specific timepoint based on genetic markers^1^. These models can be used to predict the traits in other genotypes for which genetic markers are available, thus foregoing additional measurements; the accuracy of predictions depends on representativeness of the training set and on the genetic architecture of the predicted trait^2^. As a result, GP accelerates genetic gain and reduces the need for labour-intensive field phenotyping in a test set of genotypes. While GP has been extended to allow and improve the simultaneous prediction of multiple correlated traits^3–5^, existing GP approaches have not yet addressed the problem of predicting the dynamics of multiple traits, that is, the expression of multiple traits at different timepoints across the entire period of growth of the plant.

Advances in high-throughput phenotyping (HTP) technologies, including non-destructive approaches such as hyperspectral, multispectral, fluorescence and thermal imaging, have enabled the detailed capture of plant morphometric, geometric and colourimetric traits at various growth stages and have resulted in large-scale time-resolved heterogeneous datasets on crop phenotypes^6–10^. These developments offer the possibility of predicting time-resolved traits from genetic markers, with the potential to revolutionize the GP. Recent efforts have cast the prediction of the dynamics of multiple morphometric traits as a spatio-temporal sequence prediction problem. The proposed solutions rely on deep learning approaches, such as spatio-temporal long short-term memory or memory-in-memory networks^11^, generative adversarial networks^12^ and a U-net encoder–decoder^13^. However, rather than directly predicting time-resolved crop traits, these approaches aim to predict a time series of images that are then used to infer the traits of interest. These studies do not incorporate genetic marker data, limiting their relevance in predicting multiple, time-resolved traits for unseen genotypes; furthermore, the underlying models are, presently, challenging to apply.

The time-resolved prediction of crop phenotypes can also be addressed by dynamic mode decomposition (DMD)^14^. DMD is an advanced data-driven method used in decomposing data from complex, time-dependent systems into spatio-temporal modes that provide low-dimensional description of the systems’ dynamics. These modes render DMD particularly useful for understanding and predicting the behaviour of systems characterized by high-dimensional data. DMD has been applied across different fields, including fluid mechanics, engineering, epidemiology^14^ and neuroscience^15^; however, no attempts have yet been made to apply DMD to data from HTP technologies or to combine this method with genetic markers.

Here, we combine DMD with GP to predict the genotype-specific dynamics of multiple morphometric, geometric and colourimetric crop traits using HTP data measured on a maize population of recombinant inbred lines (RILs) as well as an *Arabidopsis thaliana* diversity panel. Our approach, termed dynamicGP, showed consistent prediction accuracy for multiple traits and across multiple timepoints. When compared with a baseline GP approach, dynamicGP exhibited higher accuracies at the majority of timepoints and irrespective of traits, showing that it can effectively capture the modes and, therefore, the developmental dynamics of untested genotypes based on genetic marker data.

## Results

### DMD can predict dynamics of geometric traits in maize

DMD is a data-driven diagnostic and time-series prediction method that has found diverse applications from engineering to finance^14^ and has only recently been applied in the biological sciences^16^. To illustrate the effectiveness of DMD, we applied it to predict the dynamics of growth-related traits derived from multimodal HTP imaging measured at 25 timepoints, 5 days per week for 5 weeks, beginning at day 15 after sowing, in a maize multiparent advanced generation inter-cross (MAGIC) population of 347 RILs of which genotyping data were available for 330 (Methods and Supplementary Figs. 1 and 2). A 1-day shift of the second of three HTP experiments resulted in a 2-day gap and, thus, five consecutive measurements per week, as these were adjusted between the three experiments based on the days after sowing (DAS). By using network clustering, we selected 50 traits as representatives for the compendium of 498 top, side and combined measurements of leaf-colour and leaf-geometric traits (Methods, Table 1 and Supplementary Table 1).

**Table 1:** The representative traits for the maize MAGIC population (includes five representative traits for the maize MAGIC population as depicted in Fig. 3a–c)

Minimum	Average blue value of plant pixel colours from the top images
Q1	Average saturation of the fraction of green coloured pixels in top-view images
Mean	Mean of the saturation value of the plant pixel colours in the top-view images
Q3	Standard deviation of the blue value of the plant pixel colours in the fluorescent side-view images
Maximum	Average blue value of the plant pixel colours from the top images

**Fig. 1:**
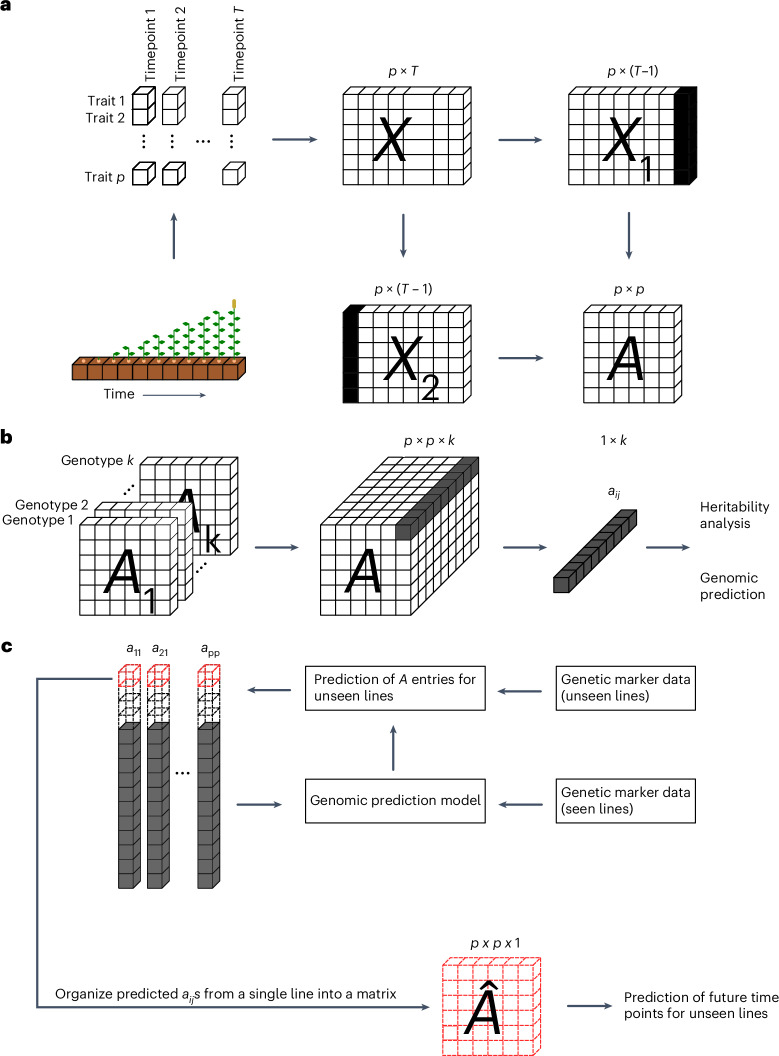
A schematic representation of dynamicGP. **a**, For a single genotype with measurements for *p* traits at *T* timepoints, we seek to find a time-invariant best-fit linear operator *A*, which transforms the phenome, given by measurements for *p* traits, at time *t* into the phenome at time *t* + 1, according to ${{{x}}}_{{{t}}{{+}}{{1}}}={{{Ax}}}_{{{t}}}$. When organized into matrix form for all timepoints, we obtain a *p* × *T* matrix *X*. Two submatrices, *X*_1_ and *X*_2_, are offset by a single timepoint and are derived from *X* by omitting the measurements at the last and the first timepoint, respectively, and are then used to calculate *A* using equation (3) of the classical DMD method in algorithm 1 (Methods). **b**, When the time-resolved data are available for *k* genotypes, we obtain a *p* × *p* × *k* tensor whose elements can be treated as traits in heritability analyses and GP. **c**, The models for the operator entries are trained and then used to obtain predictions of entries, ${\hat{a}}_{{ij}}$ (red), for unseen lines, which are then gathered into a matrix that is then used to predict future timepoints for the line.

For a single maize line, the time-resolved phenotype was arranged into a *p* × *T* matrix *X*, where *p* is the number of traits and *T* is the number of timepoints (Fig. 1a). Two submatrices, *X*_1_and *X*_2_, which are offset by a single timepoint are derived from *X* (Fig. 1a) and then used to calculate a best-fit linear *p* × *p* operator (matrix) *A*, linking the phenotype at one timepoint with the phenotype in the following timepoint (Fig. 1a). We note that the usage of linear operator does not assume that the traits truly change linearly with time. The operator *A* is usually non-symmetric and can be computed directly using the classical DMD approach (‘Algorithm 1’ section in the Methods).

We first tested how accurately the operator *A*, calculated using the data from a specific genotype, was able to recreate the underlying time-resolved phenotypes. The collection of predicted (*P*) traits, *x*_*P*,*t* + 1_, at timepoint *t* + 1 can be obtained from the multiple traits, *x*_*t*_, at timepoint *t* using the operator *A*. The essential decision one can then make is whether the multiple traits *x*_*P*,*t* + 1_ are obtained by using: (1) measured traits *x*_*t*_ at timepoint *t*, resulting in the iterative version of dynamicGP or (2) predicted traits *x*_*P*,*t*_ at timepoint *t*, resulting in the recursive version of dynamicGP, since the prediction at any timepoint *t* is obtained by recursively unfolding the process forwards from an available measurement of *x*_*t*_ at a given initial timepoint *t* = 1. Since the operator *A* captures the dynamics of multiple traits, it allows prediction of the multiple traits at any timepoint, irrespective of where it lies in the temporal expression of traits. We note that the recursive scenario is particularly relevant in applications, since it is expected to minimize the need for phenotyping.

We found that the operator *A*, derived from the classical DMD approach, was able to nearly perfectly recreate the training data when used iteratively, with a mean prediction accuracy of ~1 across all traits over the timepoints within a 5-day block of daily measurements and a mean prediction accuracy of 0.70 (±0.20) for the timepoints immediately following the 2-day gap, for which no data were available (Extended Data Fig. 2). In the recursive scenario, the classical DMD approach resulted in a perfect recreation of the training data for the first 5-day block; however, following the first 2-day gap, the prediction accuracy decreased rapidly as a result of error propagation (Extended Data Fig. 2). These results indicated that the classical DMD approach yielded models that overfitted to the training data and were not robust to slight deviations in the input data.

We additionally tested another DMD algorithm, named Schur-based DMD, which has improved numerical stability^17^. This algorithm uses the singular vectors associated with the *r* largest singular values of *X*_1_ alongside the Schur decomposition to reconstruct another operator *A*_*r*_(‘Algorithm 2’ section in the Methods), which is used in place of the operator *A* in the prediction procedure outlined above. We note that like the operator *A*, *A*_*r*_is not necessarily symmetric. In comparison with *A* from the classical DMD, we found that use of *A*_*r*_ in the iterative scenario resulted in decreased mean prediction accuracy across all traits and all timepoints of 0.84 (±0.18). However, in the recursive scenario, which is particularly relevant for practical applications, we observed robust performance, supported by the mean prediction accuracy of 0.78 (±0.16) and 0.79 (±0.13) across all traits and all timepoints and for the last timepoint, respectively (Extended Data Fig. 3). These findings demonstrate that the Schur-based DMD exhibited good predictive performance and prompted our investigation of the extent to which the elements of *A*_*r*_ and corresponding matrices used in its derivation can be predicted from genetic markers.

### The building blocks of dynamicGP are heritable

For each genotype, the operators *A* and *A*_*r*_, as well as the intermediate component matrices in the Schur-based DMD (‘Algorithm 2’ section in the Methods), can be determined from phenotypic data. Here, we treat each individual entry of these intermediate component matrices as a trait in a single-trait GP model (Fig. 1b). Our approach, termed dynamicGP, uses genetic markers as predictors and the individual matrix entries as the response variables in ridge-regression best linear unbiased prediction (RR-BLUP) models, which are collected together and organized into their respective matrices (Fig. 1c). These are, in turn, combined with selected phenotypic measurements to make longitudinal predictions of plant traits for unseen genotypes.

Here, the use of the Schur-based DMD has the advantage that its intermediate components contain fewer entries than either *A* or *A*_*r*_. This renders the components of Schur-based DMD a potential entry point for the computationally efficient prediction of *A*_*r*_ from genetic markers. We examined the marker-based heritability of the entries of all the intermediate matrices of the Schur-based DMD using 70,846 single nucleotide polymorphisms (SNPs) from the MAGIC maize population (Methods). These matrices include those of the singular value decomposition of ${{{X}}}_{{{1}}}={{U}}{{\Sigma }}{{{V}}}^{{{\mathrm{T}}}}$, the rank-reduced representation of the operator *A*, denoted by $\widetilde{{{A}}}$, the matrices *Q* and *R* resulting from its Schur decomposition as well as the projected DMD modes, *Φ* (‘Algorithm 2’ section in the Methods).

**Fig. 2:**
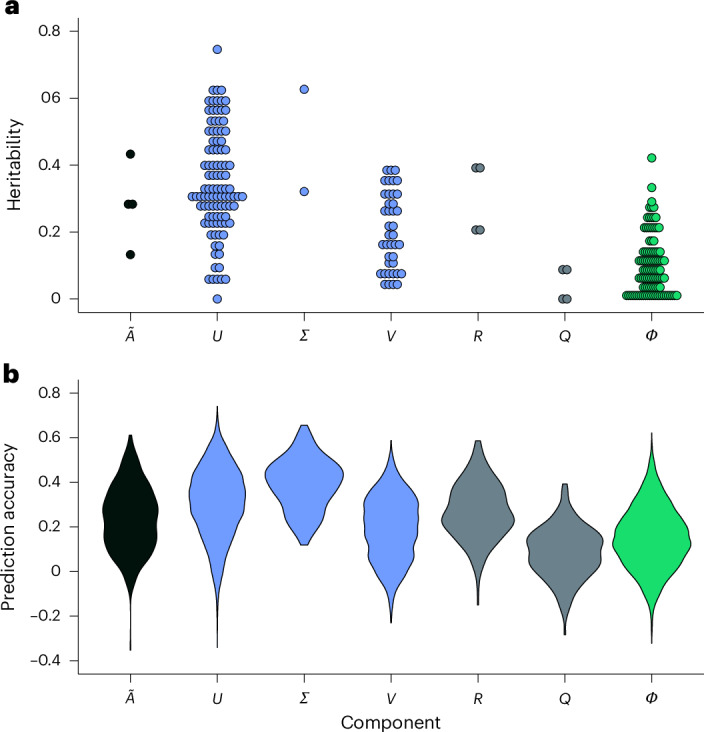
Heritability estimates and prediction accuracy of the building blocks of dynamicGP on the maize dataset. **a**, The heritability estimates for each entry of the matrices from algorithm 2 used as building blocks of dynamicGP, as obtained by genomic-relatedness-based restricted maximum likelihood (GREML). Here, $\widetilde{{{A}}}$ denotes the matrix resulting from the rank-reduced proper orthogonal decomposition-projected representation in algorithm 2; *U*, *Σ* and *V* are obtained from the singular value decomposition of *X*_1_; *R* and *Q* are matrices obtained from the Schur decomposition of $\widetilde{{{A}}}$, while *Φ* collects the projected modes from DMD of the operator *A*. **b**, The prediction accuracy of the building blocks of dynamicGP using RR-BLUP in 20 iterations of a 5-fold cross-validation. Source data

We first observed that the non-zero heritability measures for the entries of these matrices were obtained by using the first two singular vectors (*r* = 2, Methods). Specifically, we found that the mean heritability of $\widetilde{{{A}}}$ entries was 0.28, ranging from 0.13 to 0.43. Similarly, the entries of matrix *U*_*r*_ exhibited a mean heritability of 0.35, ranging from ~0 to 0.75. Moreover, the entries of *Σ*_*r*_ displayed a mean heritability of 0.47, while those of *V*_*r*_ showed a mean heritability of 0.20, ranging from 0.03 to 0.39 (Fig. 2a). Regarding the Schur decomposition of $\widetilde{{{A}}}$, the mean heritability of *R* entries was 0.30, ranging from 0.20 to 0.39. In addition, the *Q* entries demonstrated a mean heritability of 0.04, ranging from ~0 to 0.09. Notably, the mean heritability of *Φ* entries was 0.10, ranging from ~0 to 0.42 (Fig. 2a). These findings indicated that substantial parts of the building blocks of dynamicGP exhibit moderate to high heritability, suggesting that they may be predictable from genetic markers, and we can therefore use them to predict operator *A*_*r*_.

To test this hypothesis, we used 20 iterations of a 5-fold cross-validation to examine the genomic predictability of the components of the singular value decomposition of *X*_1_and the entries of $\widetilde{{{A}}}$ using SNPs. The entries of $\widetilde{{{A}}}$ exhibited a mean prediction accuracy of 0.24 (±0.15), while the entries of matrix *U*_*r*_ demonstrated a slightly higher mean accuracy of 0.31 (±0.16). Moreover, the entries of *Σ*_*r*_ showed a mean prediction accuracy of 0.39 (±0.12), whereas those of *V*_*r*_displayed a slightly lower mean accuracy of 0.19 (±0.14). Regarding the Schur decomposition of matrix $\widetilde{{{A}}}$, the entries of *R* exhibited a mean accuracy of 0.27 (±0.13), while those of *Q* showed a lower mean accuracy of 0.08 (±0.12); in addition, the mean accuracy of the entries of *Φ* was 0.15 (±0.14) (Fig. 2b). These results suggested that the building blocks of dynamicGP can indeed be predicted from genomic data using standard GP models.

### Two versions of dynamicGP differ in performance in maize

Having established that the elements of the building blocks of dynamicGP are heritable and predictable, we next used the predicted values for each entry in matrices *Φ* and *R*. Specifically, we used ten iterations of a 5-fold cross-validation with a validation step to recreate the predicted operator *A*_*r*_ for each unseen line (Fig. 1c). These predicted operators were used to make longitudinal predictions of all 50 traits over the entire time domain using the two versions of dynamicGP, iterative and recursive, defined similarly to the usage of DMD (Methods).

**Fig. 3:**
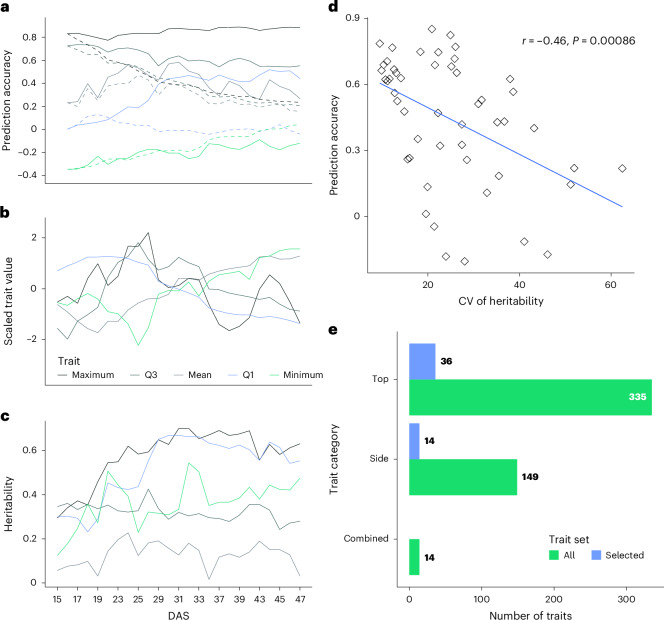
Consistency of trait heritability across time influences the prediction accuracy of dynamicGP on the maize dataset. **a**, The longitudinal prediction accuracy of representative traits corresponding to the maximum, minimum and mean, as well as the first and third interquartiles of all 50 traits across the 24 predicted timepoints (Table 1). The operator *A*_*r*_ was obtained from dynamicGP using 5-fold cross-validation (CV). The solid and dashed lines represent predictions from the iterative and recursive versions of dynamicGP, respectively. **b**, The time-resolved, scaled mean trait values across all accessions, depicting the different dynamics of traits. **c**, The heritability of the five representative traits across time (see Extended Data Fig. 1 for depiction of the dynamics of these traits). The colours for the particular traits are maintained in **a**–**c**. **d**, A Pearson correlation between the mean prediction accuracy of traits from iterative dynamicGP and the coefficient of variation of heritability estimates of traits across time (The *P*value is derived from two-sided test from a sample of 50 points). **e**, A category membership of traits in the full dataset (498 image-drived traits) compared with the traits that were selected by our clustering method for inclusion in the analysis (50 image-derived traits). Source data

We found that the iterative approach yielded more consistent accuracies across the investigated timepoints compared with the recursive approach (Fig. 3a). Across all traits and timepoints, the iterative approach resulted in a mean prediction accuracy of 0.44 (±0.32). The average blue value of plant pixel colours from top images, which quantifies the blue–yellow colour space of visible light (see Supplementary Table 1 for trait descriptions), emerged as the best-performing trait, with a mean prediction accuracy of 0.85 (±0.07). In contrast, the mean of the *a*value of plant pixel colours in side-view images showed the lowest prediction performance of −0.20 (±0.14). Conversely, the recursive approach had a mean prediction accuracy of 0.22 (±0.25) across all traits and timepoints. Here, mean of the *a*value of plant pixel colours in fluorescent top-view images could be predicted with mean prediction accuracy of 0.52 (±0.14), while the normalized fraction of pixels with mean 12% brightness in top images, as quantified in the hue, saturation and value colour space, again displayed the lowest mean prediction accuracy of −0.18 (±0.13). The recursive predictions, as expected, tended towards zero at later timepoints as prediction errors compounded over time.

We observed that traits exhibiting consistent heritability over time, indicated by smaller values for the coefficient of variation, tended to demonstrate higher prediction accuracies across all timepoints. This relationship was supported by a moderate negative Pearson correlation coefficient of −0.46 between the mean prediction accuracy for the traits from iterative dynamicGP across all timepoints and the coefficient of variation of the heritability estimates across the entire timepoints (Fig. 3d). The corresponding correlation coefficient for recursive dynamicGP was −0.41 (Supplementary Fig. 2). Therefore, in line with expectations, traits whose heritability does not vary during development were found to be better predicted by dynamicGP approach.

### DynamicGP outperforms baseline models in maize

To assess the predictive performance of dynamicGP, we compared it with a GP baseline approach using RR-BLUP models, which is the standard in breeding programmes. The RR-BLUP models were trained with data on each trait from the first timepoint in a training set and used to predict the trait in all subsequent timepoints for the testing set. Inspecting the difference of performance between dynamicGP and the baseline for traits with different predictability, we found that both the iterative and recursive versions of dynamicGP outperformed the baseline RR-BLUP models consistently across all investigated timepoints (Extended Data Fig. 4). Specifically, the mean prediction accuracy across all traits for the first timepoint, on which the models were trained, was 0.26 (±0.15). While this accuracy was maintained when the baseline RR-BLUP models were applied to predict the traits over the first three subsequent timepoints, already at the second timepoint, both versions of dynamicGP outperformed the baseline (Extended Data Fig. 4). Moreover, the iterative version of dynamicGP outperformed both the baseline and the recursive version of dynamicGP for every timepoint.

**Fig. 4:**
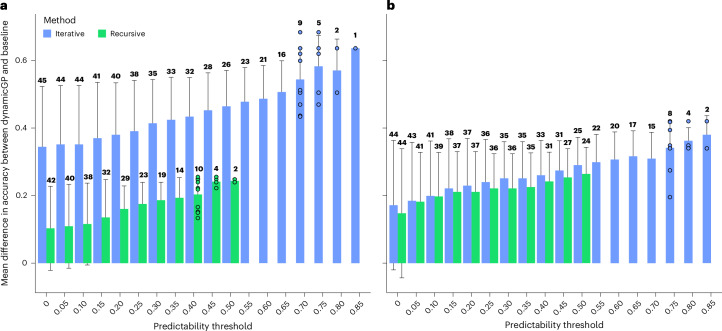
DynamicGP outperforms the baseline models on the maize dataset. **a**, The prediction of the complete time-series in iterative and recursive configurations in unseen lines. **b**, The predictions of only the last five timepoints in unseen lines in models trained with the data from the first 20 timepoints in the maize dataset. We determined the difference in performance of the iterative and recursive versions of dynamicGP and baseline RR-BLUP models, for traits whose predictability is above a threshold value, specified on the *x* axis. The number of traits with predictability larger than a specified threshold value is indicated above the respective bar. The best predicted traits with dynamicGP exhibited the greatest difference from the baseline predictions. The bars indicate the mean (±standard deviation) difference in trait prediction accuracies of traits that have mean prediction accuracies above the threshold. The number of samples (*n*) is specified on the top of each bar. Source data

We examined the differences in mean prediction accuracy across all timepoints between dynamicGP and the baseline models for each trait. The best-performing traits using the recursive method, including the mean of the *a*value of plant pixel colours in fluorescent top-view images and the mean of the *x* values in the fluorescent *xyz* colour space from top images, showed mean prediction accuracies above 0.5 of all timepoints, which was 15% higher (0.52 versus 0.45) than the best-performing trait in the baseline models (Fig. 4a). In the iterative method the best-performing traits, namely the average blue value of plant pixel colours from top images, as well as the mean of the blue value of plant pixel colours in side-view images (Supplementary Table 1), showed mean accuracies above 0.8 at all timepoints, which was 89% higher (0.85 versus 0.45) than the best trait from the baseline model predictions (two-sided *t*-test, *P* < 0.001 for all timepoints, Fig. 4a). Furthermore, both versions of dynamicGP yielded mean squared errors much lower than the RR-BLUP baselines (Extended Data Fig. 5).

We performed an additional test to determine the ability of our method to predict unseen lines in unseen timepoints (Supplementary Fig. 3, scenario 2). To this end, we trained a dynamicGP model on only the first 20 timepoints and tested its performance on the final 5 timepoints. We approached this in two ways: (1) beginning with the 1st timepoint (day 15) and predicting through to the end of the time series (day 47) and (2) beginning with the 20th timepoint (day 40) and predicting the 21st through the 25th (from day 43 to day 47). We compared these predictions with those from the RR-BLUP baseline models trained on day 15 and day 40, respectively, and used in predicting the remainder of the time series through to day 47. We observed that in both the tested configurations, iterative and recursive dynamicGP outperformed the respective baseline models (Supplementary Fig. 4). Furthermore, the two dynamicGP versions both outperformed the baseline with regards to the mean squared error (Supplementary Fig. 5) and in terms of the number of better predicted traits (Fig. 4b).

### DynamicGP can be applied to data from *A. thaliana*

To demonstrate the applicability of dynamicGP, we tested our approach with the BLUEs for 132 traits in time series consisting of 13 timepoints measured in a diversity panel of *A. thaliana* composed of 382 genotypes (experiment number 3 (EXP3) from ref. ^18^). After clustering of the traits following the same protocol as for maize, 45 traits were used in dynamicGP. We observed that although algorithms 1 and 2 performed as expected (Supplementary Fig. 6 and Extended Data Fig. 6), the matrix entries of the intermediate component matrices exhibited much lower heritabilities than the traits in the maize dataset, above, across all values for the number of components, *r* (Supplementary Figs. 7 and 8). As a result, we observed low prediction accuracies for the components of *R* and *Φ* (Extended Data Fig. 7). Despite this finding, we obtained positive prediction accuracies for some of the included traits (Extended Data Figs. 8a and 9). Note that the time-resolved heritabilities for the traits in the *A. thaliana* diversity panel were in general lower than those observed in the maize MAGIC dataset (Extended Data Fig. 8c). Interestingly, both iterative and recursive versions of our method again outperformed RR-BLUP baseline predictions in terms of the mean prediction accuracy across all traits and timepoints (Extended Data Fig. 9), mean squared error after the third timepoint (Supplementary Fig. 9) and in the number of traits with higher mean prediction accuracy across the full time series (Extended Data Fig. 10). These results further demonstrate the predictive power of dynamicGP over the baseline GP models.

## Discussion

Developing computational approaches that can predict the dynamics of crop growth and developmental plasticity for unseen genotypes in new environments holds the promise to revolutionize breeding practices. Such approaches can provide insights into how the genotype affects the time-resolved phenome and also can highlight the temporal dependence between traits comprising the phenome. While there have been important developments in GP of multiple traits^3–5^, they essentially operate with snapshot data and do not capture the dynamics of crop traits.

Here, we introduce dynamicGP, a computational approach that resolves the problem of predicting the growth dynamics across development in crops for which time-series measurements of morphometric and geometric growth-related traits for multiple genotypes are available from HTP platforms^6–10^. DynamicGP essentially applies GP to the building blocks of DMD, thus facilitating the prediction of multiple traits over time of unseen genotypes. Using a time-resolved HTP data collected for 5 days per week for 5 weeks beginning at day 15 after sowing from RILs of a maize MAGIC population, we showed that both the iterative and the recursive versions of dynamicGP outperformed the baseline GP approach. This finding was confirmed by applying dynamicGP to an independent HTP dataset from an *A. thaliana* diversity panel obtained from a different phenotyping platform.

In addition, we show that at the predictability threshold of 0.5 for two of the traits, recursive dynamicGP exhibited a better performance compared with the baseline GP approach. These traits were both top-down images measured in the fluorescence colour space. For the iterative dynamicGP, we found nine traits above a threshold of 0.7 where dynamicGP outperformed the baseline, including two traits above a threshold of 0.8, both measured in the visible colour space, namely, the average blue value of plant pixel colours from top images and the mean of blue value of plant pixel colours in side-view images (Supplementary Table 1). Further, we observed that the mean squared error of dynamicGP was lower for both the iterative and recursive versions than the baseline, with log-transformed mean squared errors of −4.30 and −3.89 for iterative and recursive, respectively, and −3.00 for the baseline (Extended Data Fig. 5). Therefore, dynamicGP yields predictions that are closer to the true values than the RR-BLUP baseline models.

The performance improvement of dynamicGP over the classical GP approach is due to the usage of time-resolved phenotypic data. While the availability of such data is rapidly increasing due to deployment of HTP in controlled environments and on the field, dynamicGP is more resource intensive than the classical GP. However, dynamicGP does not rely on model fitting (for example, of growth curves) and subsequent usage of the model parameters in GP, thus avoiding propagation of error and often unfounded assumptions about models that best describe the dynamics of single traits. Instead, dynamicGP builds on the advantages of DMD that allows the simultaneous investigation of multiple traits over time.

Although dynamicGP is applicable with any time-resolved phenotypic data on multiple traits, we tested its performance with traits retained after correlation analysis. The selection of traits was conducted to avoid the consideration of highly correlated traits that may affect predictive performance. Similar to any data-driven approach, we hypothesize that the performance of dynamicGP depends on the number of traits included and timepoints considered, which may vary between datasets. Both the recursive and iterative versions of dynamicGP offer the possibility to make predictions about plant phenotypes of unseen genotypes given measurements of specified timepoints in time, thus reducing the need for extensive temporal measurements. In this regard, while dynamicGP provides the means to overcome the challenge of predicting temporal phenomes for unseen genotypes, it inherits the limitations of GP, such as the transferability of the models to different populations and environments^2^.

Future developments of dynamicGP can rely on extensions of DMD to consider effects of environmental factors^19^. These will facilitate further refinements of the proposed approach that are expected to have very substantial impact on breeding crop varieties adapted to particular regions as well as precision agriculture. While the application of dynamicGP considered morphogeometry and physiology-related traits, future extensions to dynamicGP will consider their relationship to agronomic phenotypes, assessed longitudinally or in a single timepoint.

## Methods

### Data generation and reuse

In three independent HTP experiments, 347 RILs and nine founder lines of a MAGIC maize population^20^ were screened for natural variation in the automated HTP facility for large plants at the Leibniz Institute of Plant Genetics and Crop Plant Research in Gatersleben, Germany^21^. The panel was divided into three equal-sized subgroups and analysed in three consecutive experiments: 2113MH (116 RILs and nine founders), 2121MH (116 RILs and nine founders), and 2137MH (115 RILs and eight founders). To ensure compatibility of the data, each HTP experiment was supplemented by an additional randomly drawn overlap of 21, 21 or 27 RILs, respectively, half of which came from each of the other two experiments. The experimental design followed a complete randomized block design with three replicates per genotype (three carriers with two plants each) overlapping with three blocks corresponding to 4 of the 12 lanes of the HTP system. The image acquisition began at 15 DAS for 31 timepoints per experiment and 6 days per week. An image acquisition event was a carrier with two plants phenotyped by one image in the top view and four images in the side view at 22°, 45°, 112° and 135°. Altogether 347,740 images were taken (2113MH: 114,710 images, 91,768 side view, 22,942 top view; 2121MH: 116,840 images, 93,472 side view, 23,368 top view; 2137MH: 116,190 images, 92,952 side view, 23,238 top view). The traits were derived from images by IAP version 2.3.0 (ref. ^22^) and were analysed by alignment by DAS. A shift of the sowing date by 1 day in 2121MH allowed alignment to DAS for only 5 days per week, so that 2 days were missing each week. Consequently, the timepoints were not consecutive but were organized in 5-day blocks, with one measurement taking place each day and no measurements for 2 days over 5 weeks.

The analysed data comprised measurements for 498 traits from side, top and combined categories derived from multicolour-space image analysis^18^ (Fig. 3e and Supplementary Table 1). A linear mixed model with random effect of genotype, experiment, lane, genotype-by-experiment interaction and replicate nested in experiment was built for each timepoint. The trait heritability was calculated by the ratio between the genotype variance component and total variance component. A summation of the best linear unbiased prediction value of the genotype and fixed effect of intercept were used as the final trait values.

The genotyping data were available for 330 maize RILs consisting of 79,557 high-quality SNPs derived from SPET genotyping^23^. The missing genotyping data were imputed using Beagle 5.2 (ref. ^24^). We then removed the SNPs with a minor allele frequency <0.05, resulting in a final set of 70,846 SNPs for GP models.

The *A. thaliana* diversity panel consisted of 384 accessions phenotyped for 132 traits at 15 timepoints in the same facility as the maize and corresponds to the constant light treatment in EXP3 from an existing study^18^. The genomic data were available for 382 accessions, comprising 207,257 SNPs after filtering for a minor allele frequency smaller than 0.05. The first timepoint was removed due to the large number of missing values, leaving 14 timepoints for the analysis. The missing trait values were imputed using the R package ‘missForest’^25^.

### Clustering and trait selection

The traits were first clustered using the Mantel correlations over all genotypes and timepoints, represented as a matrix. To this end, a network was created with nodes representing traits and edges denoting intertrait Mantel correlations of a value above 0.96. Modularity clustering, as implemented in the R package ‘igraph’^26^, was used to create clusters of traits, from which the trait with the highest mean SNP-based heritability over the considered timepoints was selected as a cluster representative in the following analyses. This resulted in 50 representative traits from three categories for the maize dataset (Fig. 3e). The mean Mantel correlation between all traits was 0.63. Using this clustering method, the mean within-cluster intertrait Mantel correlation was 0.97, while the mean intercluster correlation was 0.51. The investigations of the hierarchical clustering based on the distance matrix resulting from the Mantel correlation resulted in clusters with mean within-cluster intertrait correlations approximately equal to the intercluster correlations and was therefore deemed unsatisfactory (see Supplementary Table 2 for a comparison of modularity and hierarchical clustering). The data for each trait were then minimum–maximum normalized across all the lines and timepoints and were used in modelling. The above outlined network-based clustering method was repeated on the 132 traits in the *A. thaliana* diversity panel^18^, yielding 45 unique clusters from which we selected the representative traits that were used in the subsequent analysis.

### DMD

The DMD determines a time-invariant best-fit linear operator *A* that transforms the measurements of data at one timepoint into the measurements at the following timepoint, that is,

1 $${{{x}}}_{{{t}}+{{1}}}={{A}}{{{x}}}_{{{t}}},$$

where *x* is a column vector of traits, and *A* is a *p* × *p* matrix. When the *x* vectors of all timepoints are concatenated into matrices, equation (1) becomes

2 $${{{X}}}_{{{2}}}={{A}}{{{X}}}_{{{1}}},$$

where *X*_1_ and *X*_2_ are *p* × (*T* *−* 1) matrices that are offset by a single timepoint (Fig. 1b). From *X*_1_and *X*_2_, the matrix *A* can be directly calculated using

3 $${{A}}={{{X}}}_{{{2}}}{{{{X}}}_{{{1}}}}^{\dagger },$$

where *X*_1_^†^ indicates the Moore–Penrose pseudoinverse of *X*_1_. The operator *A* allows us to predict the columns of *X* beginning with *x*_1_. In fluid mechanics *p* is often very large (that is millions), and *T* is in tens of thousands, and there are many algorithms that rely on lower-dimensional representations. We cannot use these representations since they rely on eigenvalue decomposition that renders the lower-dimensional approximation symmetric. In our case, with *p* = 50 and *T* = 25, we can compute *A* directly from the data. However, our tests demonstrated that this approach is sensitive to deviations in input data. To address these issues and to improve numerical stability, we used algorithm 2, known as the Schur-based DMD^17^, to find the modes and to obtain an approximation of *A*, denoted as *A*_*r*_, detailed below.

#### Algorithm 1

Classical DMD:

4 $${{\boldsymbol{x}}}_{{\boldsymbol{t}}+{\boldsymbol{1}}}={\boldsymbol{A}}{{\boldsymbol{x}}}_{{\boldsymbol{t}}},$$

5 $${{\boldsymbol{X}}}_{{\boldsymbol{2}}}={\boldsymbol{A}}{{\boldsymbol{X}}}_{{\boldsymbol{1}}},$$

6 $${\boldsymbol{A}}={{\boldsymbol{X}}}_{{\boldsymbol{2}}}{{{\boldsymbol{X}}}_{{\boldsymbol{1}}}}^{\boldsymbol{\dagger }},$$

#### Algorithm 2

Schur-based DMD:

**Table:** 

${{\boldsymbol{X}}}_{{\boldsymbol{1}}}={\boldsymbol{U}}{\boldsymbol{\Sigma }}{{\boldsymbol{V}}}^{{\boldsymbol{T}}}$ (7)	where equation (7) shows a singular value decomposition of ***X***_1_.
${\boldsymbol{\widetilde{{{A}}}}}={{\boldsymbol{U}}}_{{\boldsymbol{r}}}^{{{\mathbf{T}}}}{{\boldsymbol{X}}}_{{\boldsymbol{2}}}{{\boldsymbol{V}}}_{{\boldsymbol{r}}}{{\boldsymbol{\Sigma }}}_{{\boldsymbol{r}}}^{-{\boldsymbol{1}}}$ (8)	where equation (8) shows a rank-reduced representation of ***A*** projected onto the *r* POD modes of ***U***.
${\boldsymbol{\widetilde{{{A}}}}}={{\boldsymbol{Q}}}^{{{\mathbf{T}}}}{\boldsymbol{RQ}}$ (9)	where equation (9) identifies two matrices ***Q*** and ***R*** through a Schur decomposition of $\widetilde{{{A}}}$.
${\boldsymbol{\varPhi }}={{\boldsymbol{X}}}_{{\boldsymbol{2}}}{{\boldsymbol{V}}}_{{\boldsymbol{r}}}\mathop{\sum }\limits_{{\boldsymbol{r}}}^{-{\boldsymbol{1}}}{\boldsymbol{Q}}$ (10)	where equation (10) shows projected DMD modes.
${{\boldsymbol{A}}}_{{\boldsymbol{r}}}={\boldsymbol{\varPhi }}{\boldsymbol{R}}{{\boldsymbol{\varPhi }}}^{\boldsymbol{\dagger }}.$ (11)	where equation (11) shows a reconstruction of truncated ***A***_***r***_.

In a Schur-based DMD, *U*_*r*_, *V*_*r*_ and ${{{\Sigma }}}_{{{r}}}^{-{{1}}}$ denote the matrices from the singular value decomposition of *X*_1_restricted to the first *r* columns. To account for the 2-day gaps in our data, we modified our *X*_1_and *X*_2_such that *X*_1_omitted the timepoint immediately before the gap, and *X*_2_omitted the timepoint immediately following the gap so that the difference between the columns of the two matrices was always a single time step. An R implementation of algorithms 1 and 2 is available at https://github.com/dobby978/dynamicGP.

### Heritability and GP analyses

To quantify the extent to which the selected traits and all the elements of all matrices defined in algorithm 2 can be predicted by genetic markers, we calculated the SNP-based heritability using genome-wide complex trait analysis (GCTA)^27^. The genomic relatedness matrix was generated using TASSEL 5 (ref. ^28^). We then investigated the accuracy of the GP using a RR-BLUP model, as implemented in the R package ‘rrBLUP’^29^, to predict individual elements of these matrices. The prediction accuracy for the elements was quantified as the mean Pearson correlation coefficient between the true and predicted values in 20 iterations of 5-fold cross-validations.

### Selection of the *r* value in Schur-based DMD

The number of singular values *r* was selected by analysing the heritability and predictability of components in a Schur-based DMD using $r\in \left[2,\,3,\,4,\,5,\,6\right]$. The heritabilities of the singular values and entries of the first two singular vectors are relatively high (0.47 and 0.35 on average, respectively; Supplementary Fig. 10). Most entries of the singular vectors associated with the smaller singular values exhibited near-zero heritability (Supplementary Fig. 10). For the Schur decomposition of $\widetilde{{{A}}}$ with *r* = 2 (${\widetilde{{{A}}}}_{{{2}}}$), the entries of *Q* and *R* exhibited a non-zero heritability (0.04 and 0.29 on average, respectively), while the entries of the projected DMD modes, *Φ*, had a mean heritability of 0.10 (Supplementary Fig. 11). We observed similar findings regarding to predictability that the mean prediction accuracies for the first two singular values and the entries of the singular vectors as well as of $\widetilde{{{A}}}$ were at least 0.1, while for other values of *r*, the prediction accuracy for the entries of ${\widetilde{{{A}}}}_{{{r}}}$ were near zero (Supplementary Fig. 12). Similarly, the entries of the matrices *Q* and *R* obtained from ${\widetilde{{{A}}}}_{{{2}}}$ showed a non-zero prediction accuracy, while *Q* and *R* of ${\widetilde{{{A}}}}_{{{r}}}$, with *r* larger than 2, exhibited a near-zero prediction accuracy. The matrix *Φ* had a non-zero mean prediction accuracy for an *r* value of, at most, 3 (Supplementary Fig. 13).

As *r* is a hyperparameter that must be selected before training any machine learning models, we additionally tested the heritability of the matrix elements in only the training–testing set of a training–testing–validation configuration (Supplementary Fig. 3, scenario 1). In these tests, we again observed that the matrix elements corresponding to the first two singular vectors, suggesting that this method of selection of *r* holds when the lines in the validation set are removed (Supplementary Figs. 14 and 15). Therefore, we used the first two singular vectors (*r* = 2) in the subsequent analysis, as the matrix entries corresponding to larger values of *r* were not heritable nor predictable with sufficient accuracy to contribute any useful information. This also had the benefit of acting as a noise filter, which reduced overfitting. The performance of DMD algorithm 2 across all timepoints with a different number of singular vectors retained is shown in Supplementary Fig. 16.

### Implementation of dynamicGP

The predicted (P) *Q* and *R* matrices, denoted *Φ*_P_ and *R*_P_, for each line were created by collecting the entries obtained from the RR-BLUP models and placing them in their corresponding location in the matrices; *Φ*_P_ and *R*_P_were then used in

12 $$\displaystyle{{{A}}}_{{{{\mathrm{P}}}}}={{{\varPhi }}}_{{{{\mathrm{P}}}}}{{{R}}}_{{{{\mathrm{P}}}}}{{{{\varPhi }}}_{{{{\mathrm{P}}}}}}^{\dagger },$$

to obtain a prediction of *A*_*r*_, defined in algorithm 2 (equation (8)). For each iteration of the 5-fold cross-validation, we predicted the *A*_P_ operators in the testing fold using the model from data of the training folds. The *A*_P_ operators were then used to predict the trait values in testing fold. The accuracies of prediction were assessed for each trait using the Pearson correlation between the predicted trait values and the measured trait values. This resulted in 100 predictions of each trait at each timepoint, and the mean value was reported as the final predication accuracy.

### Iterative and recursive versions of dynamicGP

Trait values in the following timepoints were predicted in two scenarios, namely iterative and recursive. In the iterative version of dynamicGP, we used the measured trait values at each timepoint to predict the values at the following timepoint, that is,

13 $$\displaystyle{{{x}}}_{{{{\mathrm{P}}}},{{t}}+{{1}}}={{{A}}}_{{{{\mathrm{P}}}}}{{{x}}}_{{{t}}}.$$

In the recursive version of dynamicGP, we used the measured trait values at *t* = 1 and then used the predicted values to predict the next timepoint over the time interval of interest, namely

14 $$\begin{array}{cccc}\displaystyle{{{x}}}_{{{{\mathrm{P}}}},{{t}}+{{1}}} & = & {{{A}}}_{{{{\mathrm{P}}}}}{{{x}}}_{{{t}}} & {\rm{for}}\,\,t=1,\\ {{{x}}}_{{{{\mathrm{P}}}},{{t}}+{{1}}} & = & {{{A}}}_{{{{\mathrm{P}}}}}{{{x}}}_{{{{\mathrm{P}}}},{{t}}} & {\rm{for}}\,\,t > 1.\end{array}$$

### Reporting summary

Further information on research design is available in the Nature Portfolio Reporting Summary linked to this article.

## Supplementary information

Supplementary Information Supplementary Figs. 1–16.

Reporting Summary

Supplementary Tables 1–4 Supplementary Table 1. A description of the maize traits included in the analysis, with the representative traits highlighted. Supplementary Table 2. A comparison of hierarchical versus Madularity clustering. Supplementary Table 3. A description of the *Arabidopsis* traits included in analysis, with the representative traits highlighted. Supplementary Table 4. The representative traits depicted in Extended Data Fig. 8.

## Source data

Source Data Fig. 2 Heritabilities and RR-BLUP prediction accuracies of matrix entries of Schur DMD matrices.

Source Data Fig. 3 Time-resolved dynamicGP accuracies, trait values and heritabilities and CV of heritability and mean prediction accuracy across the time series and trait categories.

Source Data Fig. 4 Mean differences between RR-BLUP and dynamicGP at difference thresholds, as well as points indicating trait-specific differences when *n* < 10.

**Extended Data Fig. 1:**
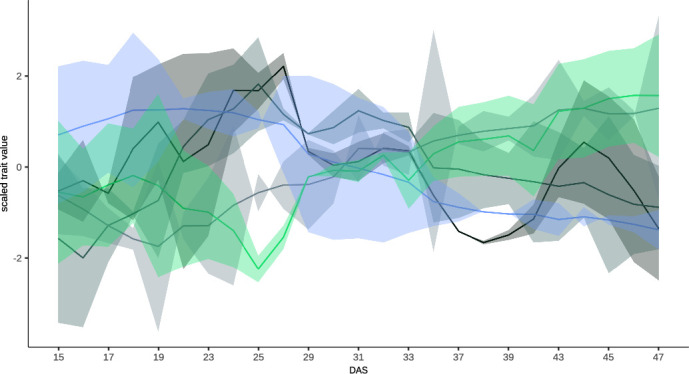
Time-resolved dynamics of five selected traits from the maize HTP data set. Expanded version of Fig. 3b, showing time-resolved, scaled mean trait values across all lines, depicting the different dynamics of traits with standard deviation depicted as shaded area. Source data

Source Data Extended Data Fig. 1 Dynamics of selected traits across the time series, including standard deviation.

**Extended Data Fig. 2:**
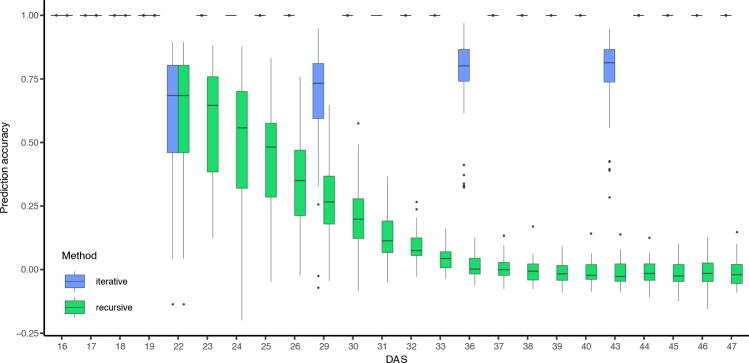
Performance of DMD Algorithm 1 on the maize MAGIC data set. The operator ${\bf{A}}$ recreates training data near perfectly for the first week. However, after the first two-day gap, past time point 5, the recursive model (green) began to accrue errors and tended towards 0 over the subsequent time points. The performance of the iterative model was unaffected aside from the days immediately following the two-day gap (after *t* = [5, 10, 15, 20]). Prediction accuracy represents the mean accuracy across 50 traits using the operator ${\bf{A}}$ calculated based on Algorithm 1, similar to that presented in Fig. 1. Horizontal lines denote the median, boxes indicate the interquartile range (IQR), whiskers indicate the extended range of 1.5× IQR. Source data

Source Data Extended Data Fig. 2 Accuracies of all traits at all timepoints after applying algorithm 1.

**Extended Data Fig. 3:**
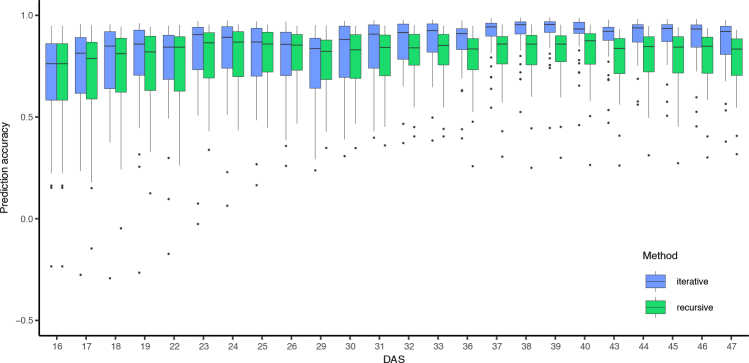
Performance of DMD Algorithm 2 on the maize MAGIC data set. Algorithm 2 filters noise in the data and increases the prediction accuracy for time points following the two-day gaps (after $t=\left[5,\,10,\,15,\,20\right]$) using the recursive approach (green). Shown is the mean accuracy across 50 traits using the operator ${{\bf{A}}}_{{\bf{r}}}$ calculated based on Algorithm 2 with the number of singular vectors, $r$, included in the truncated model fixed to a value of two. Horizontal lines denote the median, boxes indicate the interquartile range (IQR), whiskers indicate the extended range of 1.5× IQR. Source data

Source Data Extended Data Fig. 3 Accuracies of all traits at all timepoints after applying algorithm 2.

**Extended Data Fig. 4:**
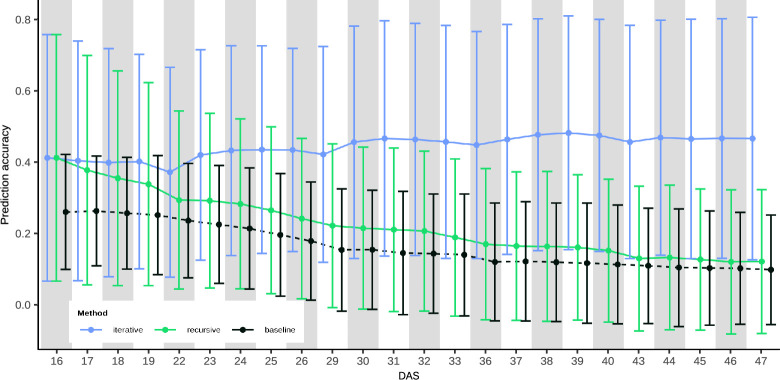
Qualitative comparison of dynamicGP and baseline over all time points in a validation configuration for the maize MAGIC data set. The iterative version of dynamicGP (blue) yields mean prediction accuracies over all traits that are greater than the equivalent prediction accuracies from recursive dynamicGP (green) in 10 iterations of nested 5-fold cross validation with a validation step. Both versions of dynamicGP outperformed RR-BLUP baselines (black) at all time points. Lines represent mean model performance across all traits and error bars denote standard deviations. Source data

Source Data Extended Data Fig. 4 Aggregated accuracies of all traits at all timepoints after applying dynamicGP with a validation and RR-BLUP benchmark.

**Extended Data Fig. 5:**
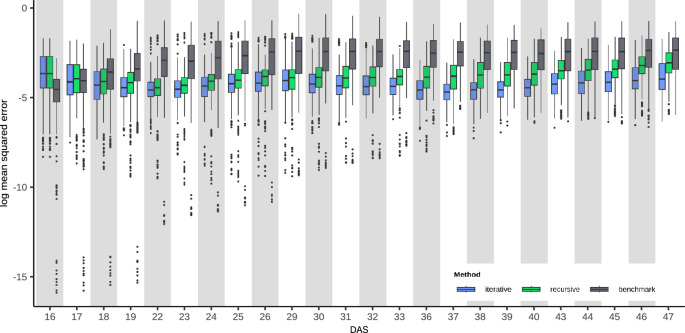
Quantitative comparison of dynamicGP and baseline over all time points in a validation configuration for the MAGIC maize data set. The iterative version of dynamicGP (blue) yields mean mean squared error (MSE) over all traits that are lower than the equivalent MSEs in recursive dynamicGP (green) in 10 iterations of nested 5-fold cross validation with a validation step. The baseline models yield lower MSEs across all traits for the first two time points, however both versions of dynamicGP outperformed the baselines (gray) at all subsequent time points. Horizontal lines denote the median, boxes indicate the interquartile range (IQR), whiskers indicate the extended range of 1.5× IQR. Source data

Source Data Extended Data Fig. 5 Mean squared error between the true and predicted values of all traits at all timepoints after applying dynamicGP with validation and RR-BLUP benchmark.

**Extended Data Fig. 6:**
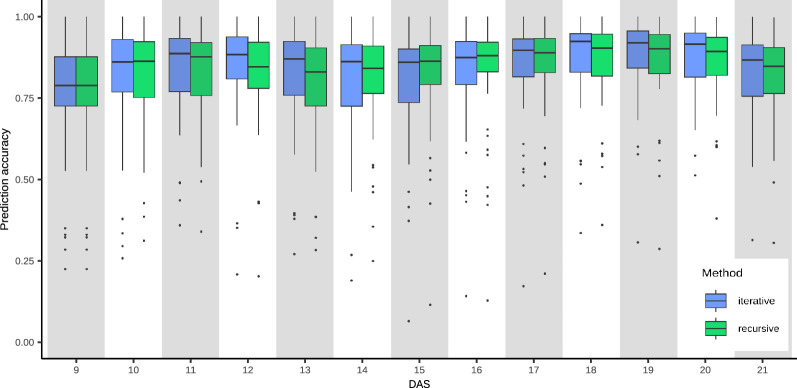
Performance of DMD Algorithm 2 on data from an *A. thaliana* diversity panel. Shown is the mean accuracy across 45 traits using the operator ${{\bf{A}}}_{{\bf{r}}}$ calculated based on Algorithm 2 with the number of singular vectors, $r$, included in the truncated model fixed to a value of two. Horizontal lines denote the median, boxes indicate the interquartile range (IQR), whiskers indicate the extended range of 1.5× IQR. Source data

Source Data Extended Data Fig. 6 Accuracies of all traits at all timepoints after applying algorithm 2 to the *Arabidopsis* dataset.

**Extended Data Fig. 7:**
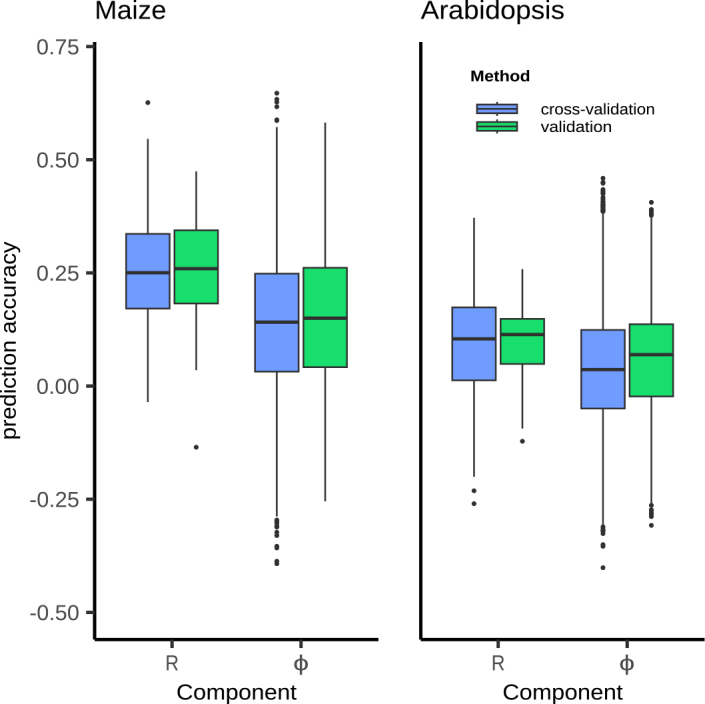
Prediction accuracy of the elements of R and Φ in data sets of an *A. thaliana* diversity panel and a maize MAGIC population. Prediction accuracy of the elements of **R** and ${\boldsymbol{\Phi }}$ after 10 iterations of 5-fold cross-validation with a validation step for Maize and Arabidopsis data sets. Horizontal lines denote the median, boxes indicate the interquartile range (IQR), whiskers indicate the extended range of 1.5× IQR. Source data

Source Data Extended Data Fig. 7 RR-BLUP prediction accuracies of matrix entries of *R* and *ϕ* for maize and *Arabidopsis*.

**Extended Data Fig. 8:**
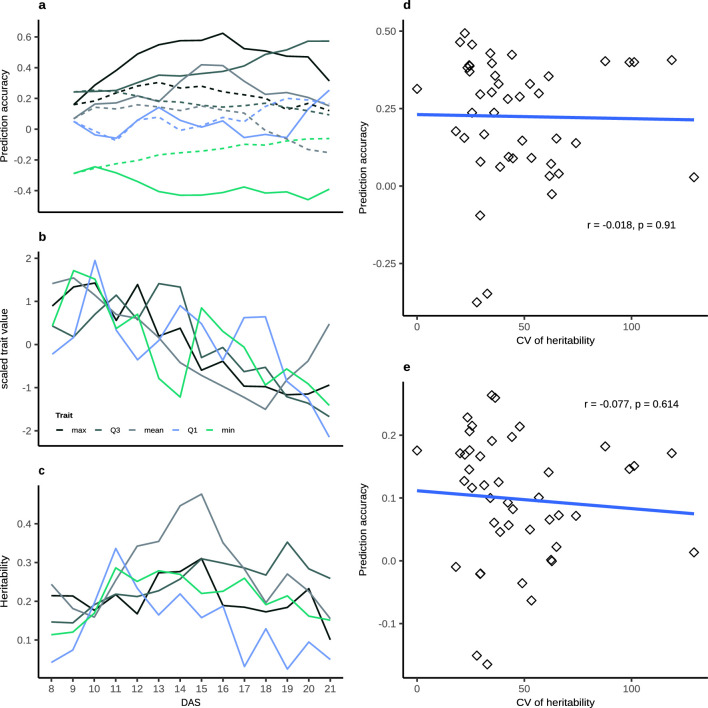
Consistency of trait heritability over time less predictive of prediction accuracy on a data set of an *A. thaliana* diversity panel. **a**. Longitudinal prediction accuracy of representative traits corresponding to the maximum, minimum, mean, as well as the first and third interquartiles of all 45 traits across the 24 predicted time points (Supplementary Tables 3 and 4). The operator ${{\bf{A}}}_{{\bf{r}}}$ was obtained from dynamicGP using 5-fold cross validation. The solid and dashed lines represent predictions from the iterative and recursive versions of dynamicGP, respectively. Colors for particular traits are maintained in panels **a** - **c**. **b**. Time-resolved, scaled mean trait values across all accessions, depicting the different dynamics of traits. c. Heritability of the five representative traits across time (p-value is derived from two-sided test from a sample of 50 points). **d** - **e**. Pearson correlation between the mean prediction accuracy of traits from iterative (**d**) and recursive (**e**) dynamicGP and the coefficient of variation of heritability estimates of traits across time. Source data

Source Data Extended Data Fig. 8 Time-resolved dynamicGP accuracies, trait values and heritabilities and CV of heritability and mean prediction accuracy across the time series for *Arabidopsis*.

**Extended Data Fig. 9:**
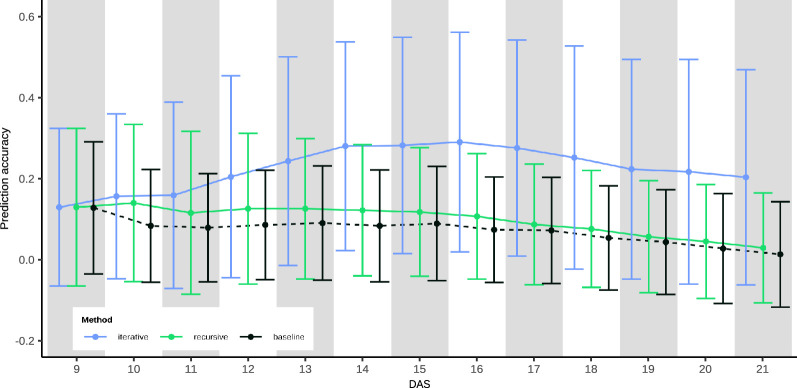
Iterative dynamicGP outperformed recursive and baseline models over all time points on a data set of an *A. thaliana* diversity panel in a validation configuration. Despite lower prediction accuracy of **R** and ${\boldsymbol{\Phi }}$, non-zero prediction accuracy was achieved for both iterative and recursive dynamicGP in a validation configuration. The iterative version of dynamicGP (blue) yields mean prediction accuracies over all traits that are greater than the equivalent prediction accuracies recursive dynamicGP (green) in 10 iterations of nested 5-fold cross validation with a validation step. Both versions of dynamicGP outperformed RR-BLUP baselines (black) at all time points. Lines represent mean model performance across all traits and error bars denote standard deviations. Source data

Source Data Extended Data Fig. 9 Aggregated accuracies of all traits at all timepoints after applying dynamicGP with validation and RR-BLUP benchmark for *Arabidopsis*.

**Extended Data Fig. 10:**
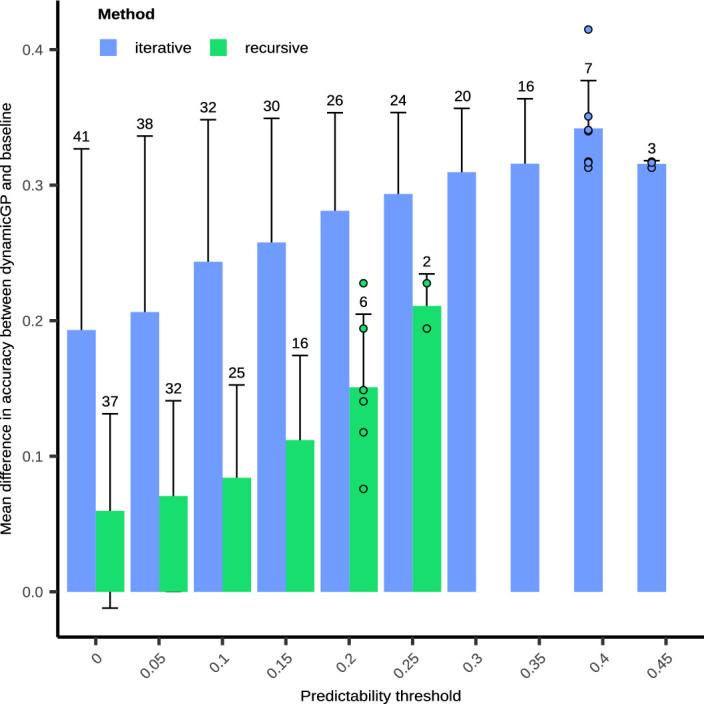
DynamicGP outperforms the baseline models on the *A. thaliana* data set. We determined the difference in performance of the iterative and recursive versions of dynamicGP and baseline RR-BLUP models, for traits whose predictability is above a threshold value, specified on the x-axis. The number of traits with predictability larger than a specified threshold value is indicated above the respective bar. The best predicted traits with dynamicGP exhibited the greatest difference from the baseline predictions. Bars indicate the mean (± SD) difference in trait prediction accuracies of traits which have mean prediction accuracies above the threshold. Source data

Source Data Extended Data Fig. 10 Mean differences between RR-BLUP and dynamicGP at difference thresholds, as well as points indicating trait-specific differences when *n* < 10.

## Data Availability

A panel of 347 MAGIC maize lines and their 9 founder lines were phenotyped at high throughput in the tier 1 phase of the CAPITALISE project^30^. The *A. thaliana* phenotyping dataset corresponds to EXP3 obtained from a published study^31^. The genotyping data for maize and *A. thaliana* are available via Zenodo at 10.5281/zenodo.14959484 (ref. ^32^). Source data are provided with this paper.

## Extended data

are available for this paper at 10.1038/s41477-025-01986-y.

## Code availability

All code that was used to generate the results of this study is available via GitHub at https://github.com/dobby978/dynamicGP and via Zenodo at 10.5281/zenodo.14959484 (ref. ^32^).
