# Supplementary material for: Predicting plant trait dynamics from genetic markers
Source: Nat Plants. 2025 Apr 17;11(5):1018–27. doi: 10.1038/s41477-025-01986-y (PMC12095066; doi:10.1038/s41477-025-01986-y)
Supplement: Supplementary file 1 — Supplementary Figs. 1–16. [file 41477_2025_1986_MOESM1_ESM.pdf]

nature plants

Supplementary information https://doi.org/10.1038/s41477-025-01986-y

# Predicting plant trait dynamics from genetic markers

In the format provided by the authors and unedited

# SUPPLEMENTARY FIGURES

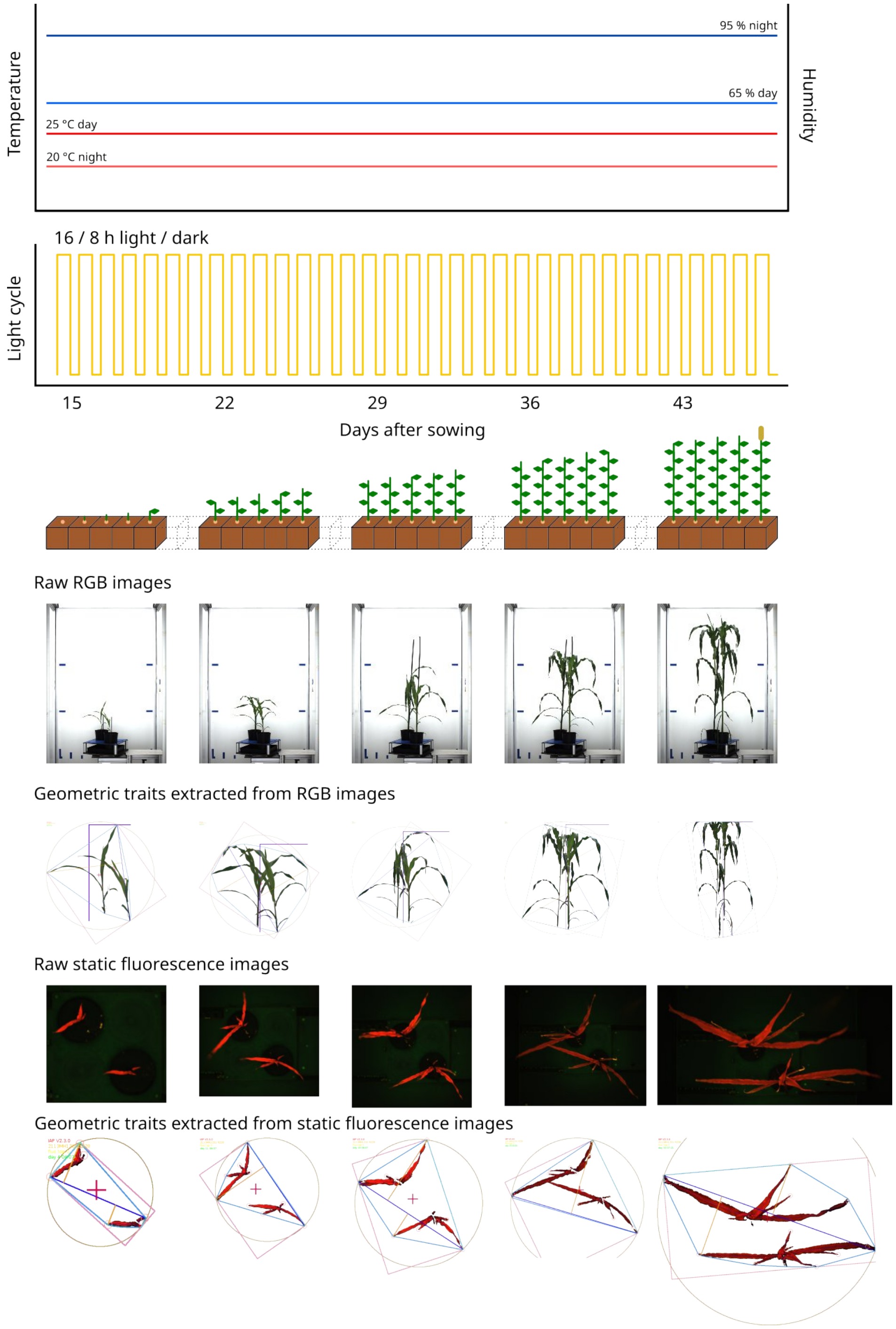


**Supplementary Figure 1. Experimental outline for maize.** Plants were grown in the IPK's automated high-throughput phenotyping (HTP) system for large plants under conditions of consistent daily fluctuations in light temperature and humidity (see Methods). Images from [10.5447/ipk/2025/0](10.5447/ipk/2025/0).

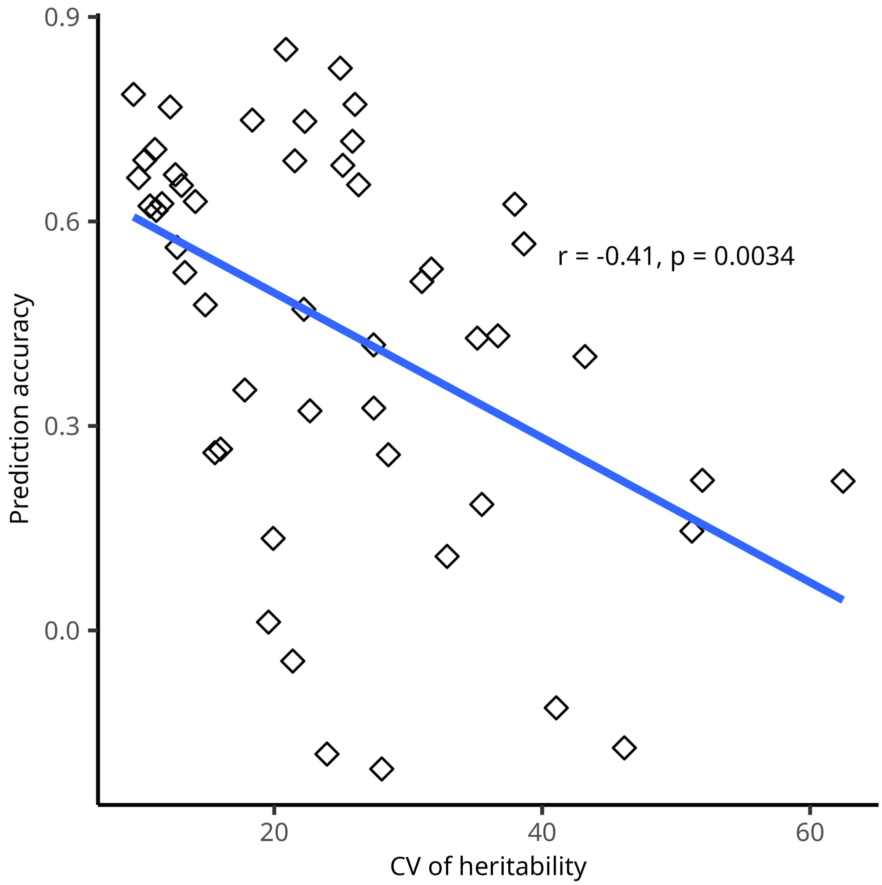


**Supplementary Figure 2. Relationship between prediction accuracy and heritability on the maize MAGIC data set.** Pearson correlation between the mean prediction accuracy of traits from recursive dynamicGP and the coefficient of variation of heritability estimates across time for all traits used in dynamicGP.

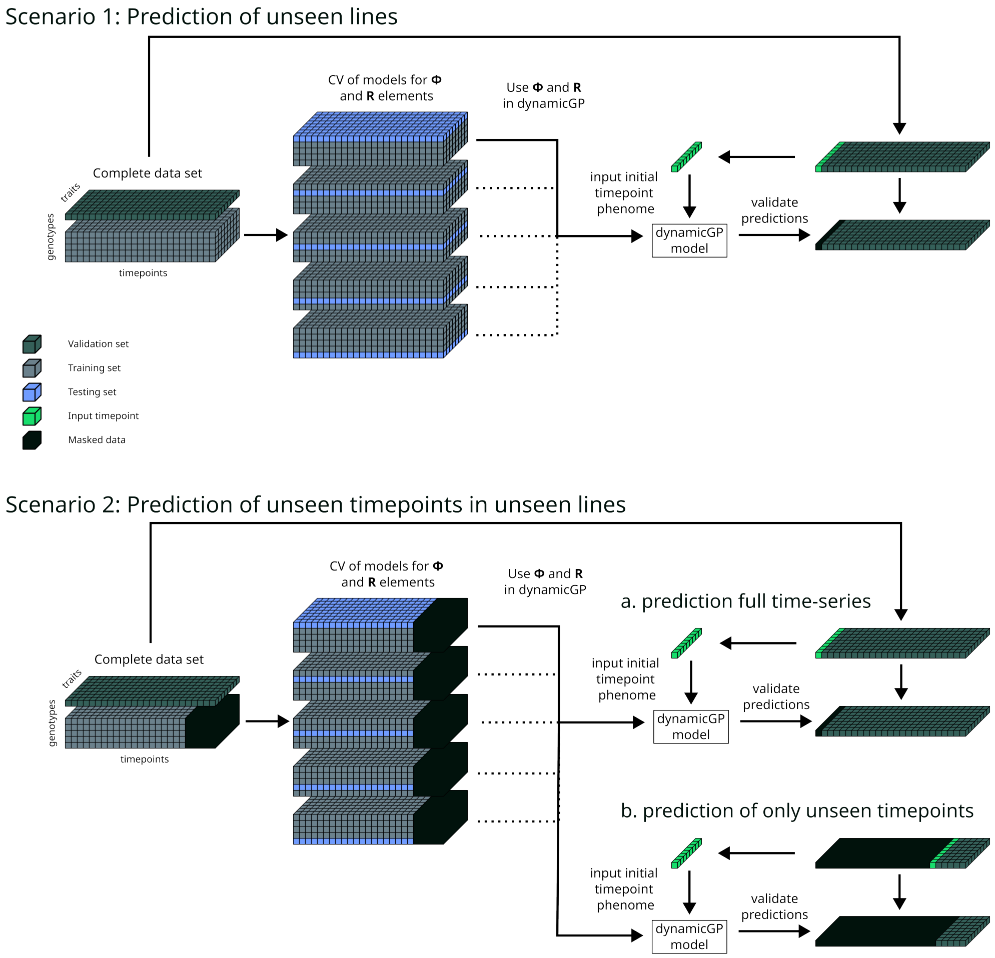


**Supplementary Figure 3. Outline of data partitioning for training-testing-validation cross-validation.** Depicted is the data use for recursive dynamicGP beginning with the first time point. The training-testing-validation was repeated 10 times, with differently randomized folds in each iteration. Scenario 1 refers to the case when all data for the lines in the training set are used for training dynamicGP, and allows investigating the prediction performance of seen time points for unseen lines. In contrast, scenario 2 denotes the case when the a subset of time points from the end of the time-series are not used to train dynamicGP, and allows investigating the prediction performance of unseen time points for unseen lines.

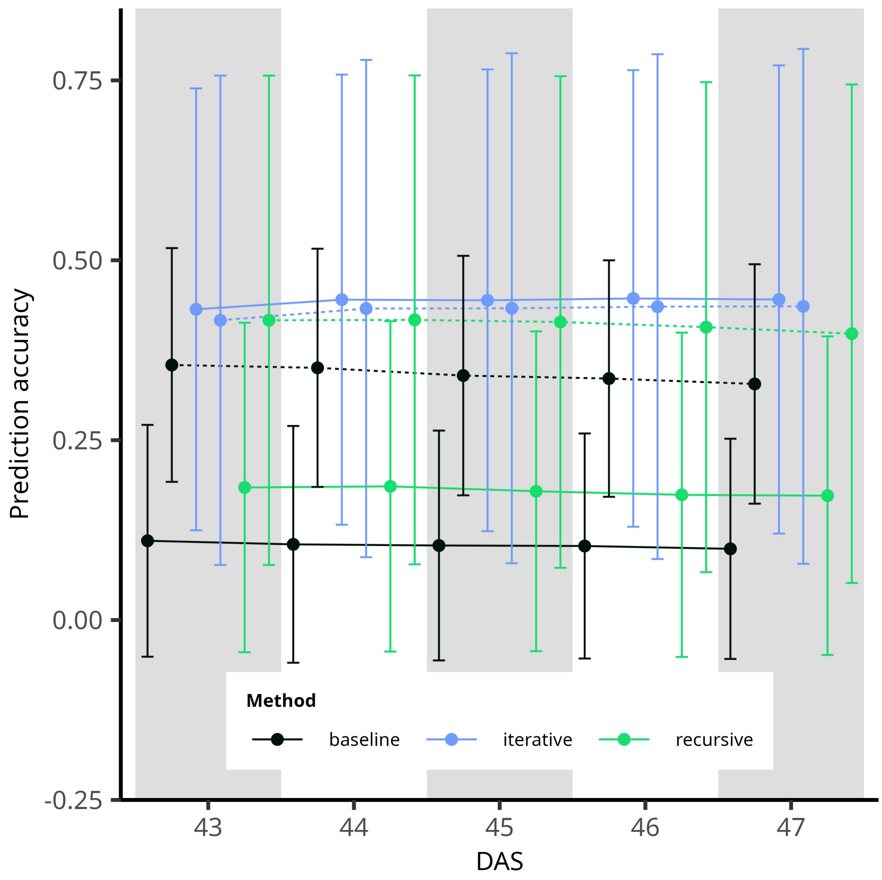


**Supplementary Figure 4**. **DynamicGP accurately predicts unseen time points in unseen lines on the maize MAGIC data set.** When the final week is left out of model training, the resulting models are still able to predict the remaining five days better than the RR-BLUP baselines (black) in both iterative (blue) and recursive (green) configurations. When given data from day 40 as a "primer", both versions of dynamicGP (dashed blue and green lines), outperform RR-BLUP baselines (dashed black line) trained on data from day 40 and used to predict the remaining time points. Lines represent mean model performance across all traits and error bars denote standard deviations. DAS denotes days after sowing.

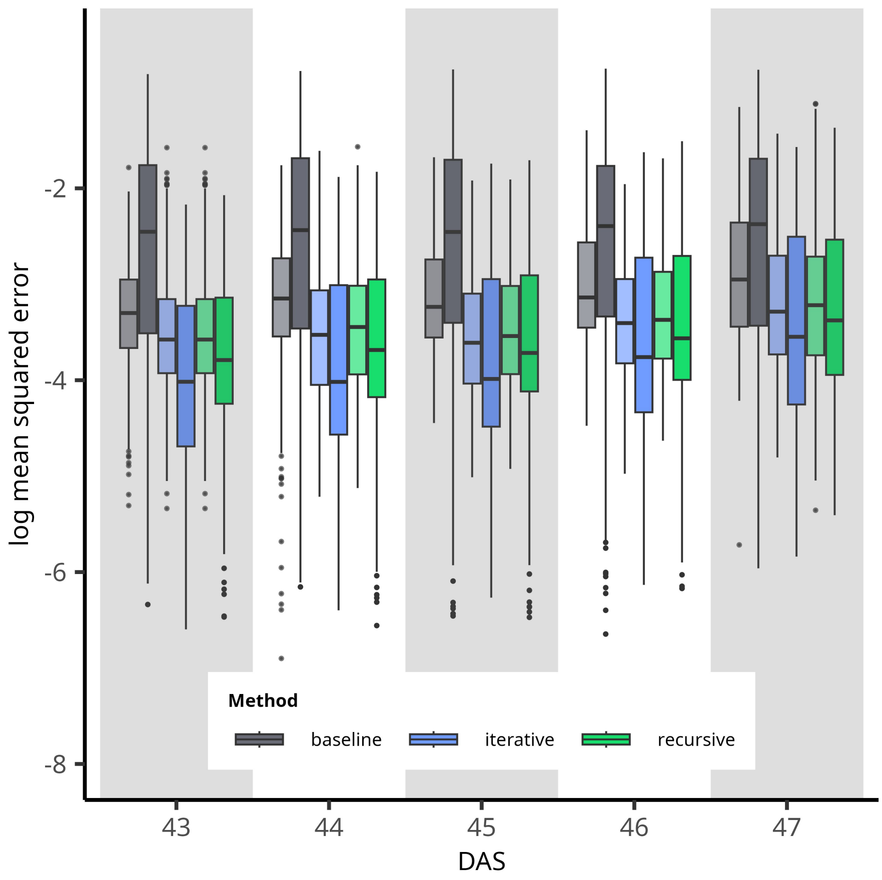


**Supplementary Figure 5. DynamicGP achieves lower mean squared error in unseen time points than RR-BLUP baselines on the maize MAGIC data set.** When the final week is left out of model training, the resulting models are still he iterative version of dynamicGP (blue) yields mean mean squared error (MSE) over all traits that are lower than the equivalent MSEs in recursive dynamicGP (green) in 10 iterations of nested 5 fold cross validation with a validation step. The baseline models yield lower MSEs across all traits for the first timepoint, however both versions of dynamicGP outperformed the baselines (grey) at all subsequent time points. When given data from day 40 as a “primer”, both versions of dynamicGP (faded blue and green boxes), outperform RR-BLUP baselines (faded gray boxes) trained on data from day 40 and used to predict the remaining time points. Horizontal lines denote the median, boxes indicate the interquartile range (IQR), whiskers indicate the extended range of 1.5× IQR. DAS denotes days after sowing.

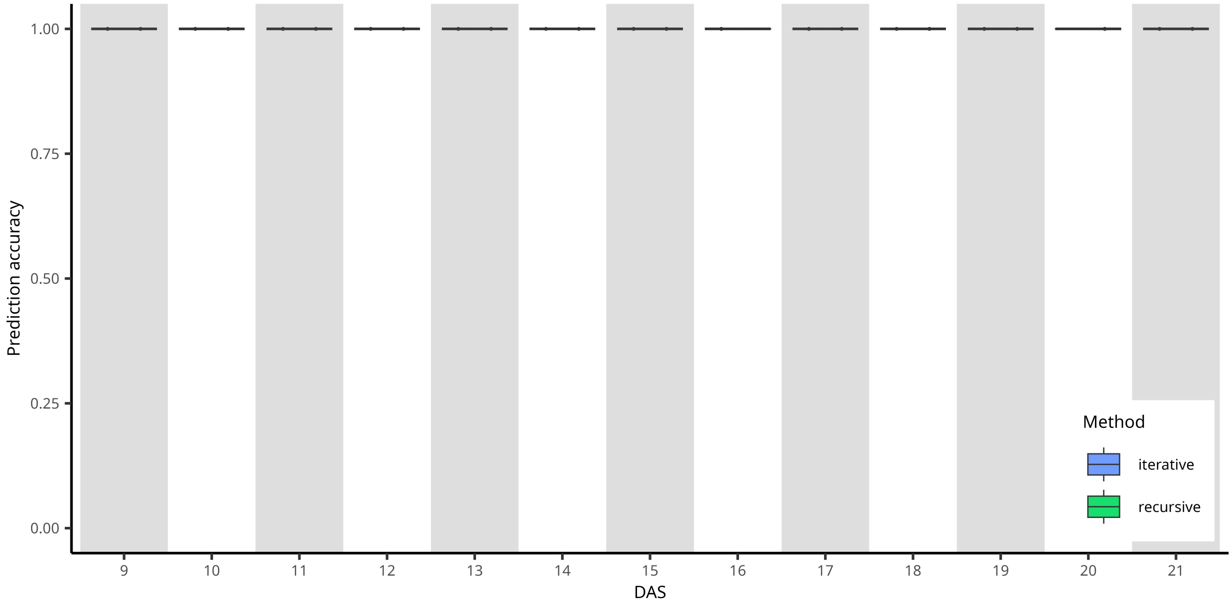


**Supplementary Figure 6**. **Performance of DMD Algorithm 1 on data from an *A. thaliana* diversity panel**. The operator **A** recreates training data near perfectly across the full time series. Prediction accuracy represents the mean accuracy across 45 traits using the operator **A** calculated based on Algorithm 1, similar to that presented in Figure 1. Horizontal lines denote the median, boxes indicate the interquartile range (IQR), whiskers indicate the extended range of 1.5× IQR.

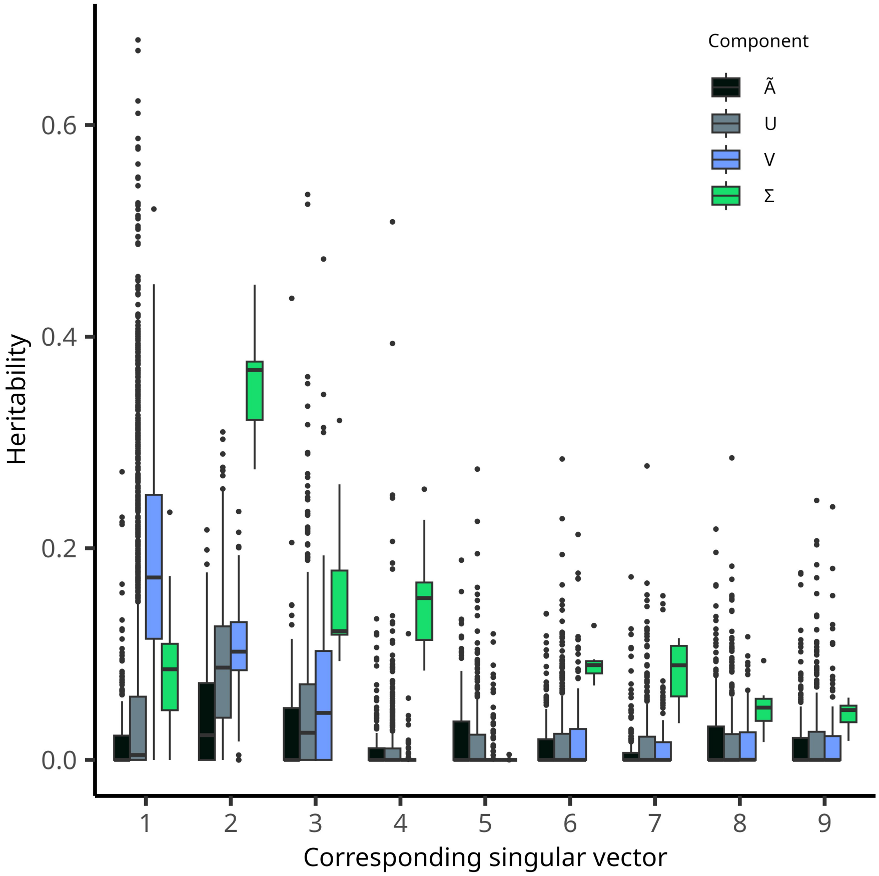


**Supplementary Figure 7 Heritability of elements of $\tilde{\mathbf{A}}$ as well as the singular values and singular vector elements of $\mathbf{X}_1$ derived from a data set of an *A. thaliana* diversity panel in a validation configuration.** The heritability of vector elements, except for $\mathbf{\Sigma}$, decreases to nearly zero after the first three singular values (SVs) in 10 iterations of training sets. Horizontal lines denote the median, boxes indicate the interquartile range (IQR), whiskers indicate the extended range of 1.5× IQR.

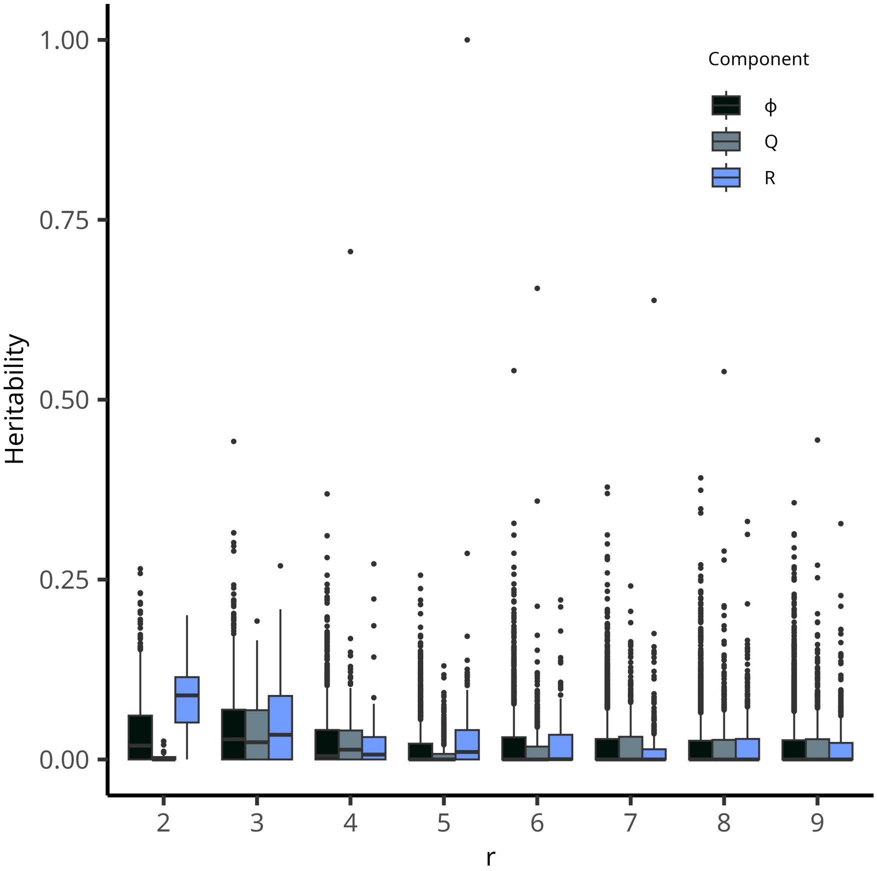


**Supplementary Figure 8**. **Heritability of the elements of Schur decomposition components and projected DMD modes on a data set of an *A. thaliana* diversity panel in a validation configuration.** The heritability of the elements of Schur decomposition components of $\tilde{\mathbf{A}}$ **(Q** and **R)** and the elements of the projected DMD modes (**Φ**) was very low for all $r$, especially $r > 3$, where $r$ represents the number of singular vectors included in the truncated model obtained from Algorithm 2. Horizontal lines denote the median, boxes indicate the interquartile range (IQR), whiskers indicate the extended range of 1.5× IQR.

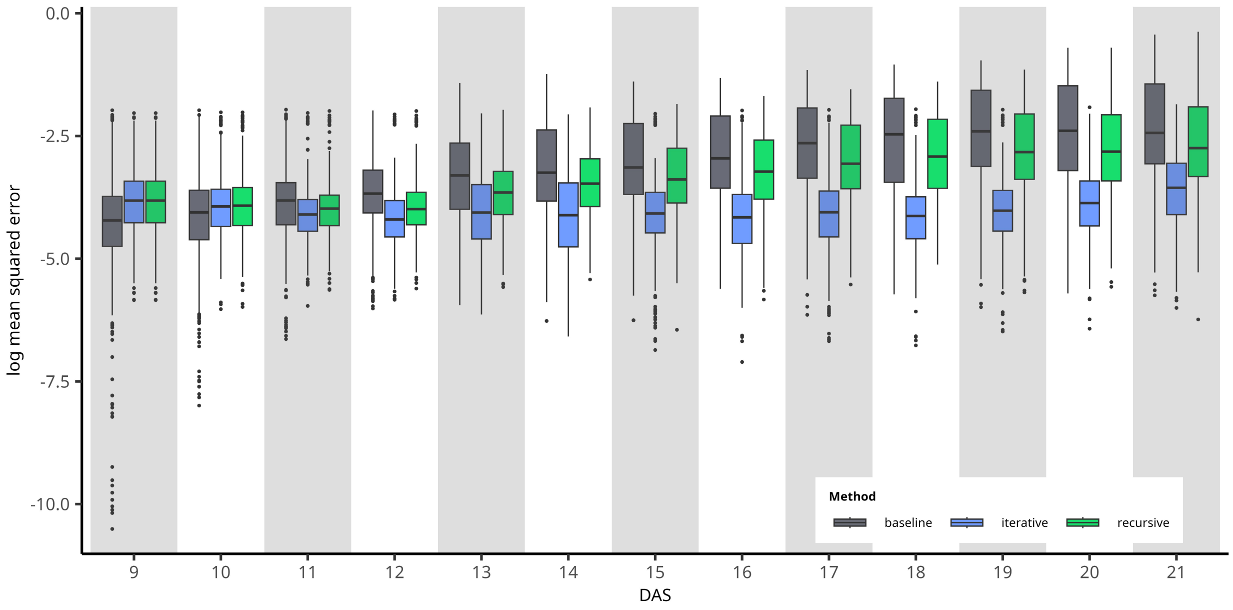


**Supplementary Figure 9**. **Comparison of iterative dynamicGP with recursive and baseline models in a validation configuration on a data set of an *A. thaliana* diversity panel.** The iterative version of dynamicGP (blue) yields mean mean squared error (MSE) over all traits that are lower than the equivalent MSEs in recursive dynamicGP (green) in 10 iterations of nested 5-fold cross validation with a validation step. The baseline models yield lower MSEs across all traits for the first two time points, however both versions of dynamicGP outperformed the baselines (grey) at all subsequent time points. Horizontal lines denote the median, boxes indicate the interquartile range (IQR), whiskers indicate the extended range of 1.5× IQR.

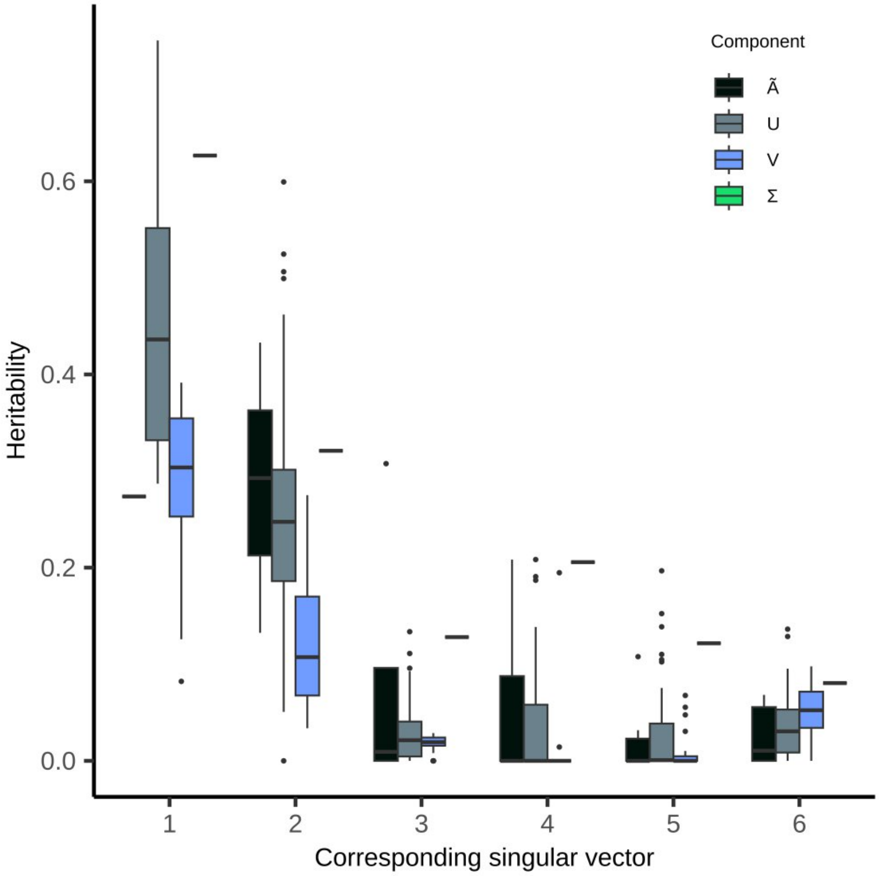


**Supplementary Figure 10. Heritability of elements of $\tilde{\mathbf{A}}$ as well as the singular values and singular vector elements of $\mathbf{X}_1$ on a data set of an *A. thaliana* diversity panel.** The heritability of vector elements decreases to nearly zero after the first two singular values (SVs). The number of singular values and vectors with heritability greater than zero provides a mechanism for selection of $r$, denoting the number of included singular vectors in the truncated model obtained from Algorithm 2. Horizontal lines denote the median, boxes indicate the interquartile range (IQR), whiskers indicate the extended range of 1.5× IQR.

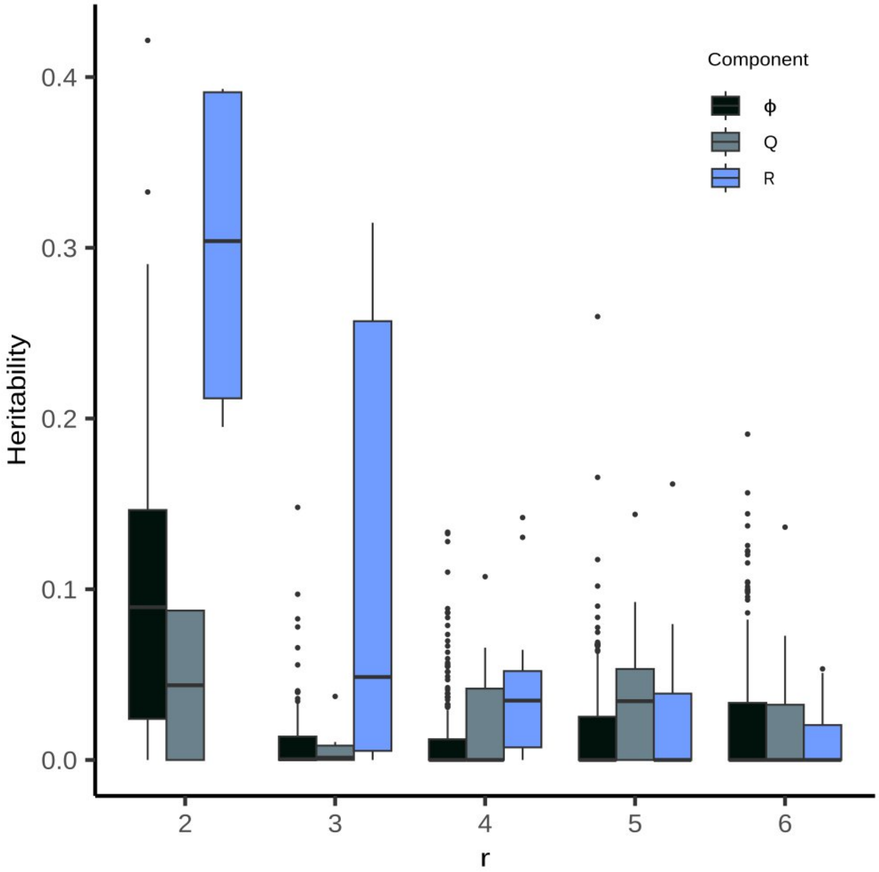


**Supplementary Figure 11**. **Heritability of the elements of Schur decomposition components and projected DMD modes on a data set of an *A. thaliana* diversity panel.** The heritability of the elements of Schur decomposition components of $\tilde{\mathbf{A}}$ **(Q** and **R)** and the elements of the projected DMD modes ($\mathbf{\Phi}$) decreased to nearly zero for $r > 2$, where $r$ represents the number of singular vectors included in the truncated model obtained from Algorithm 2. Horizontal lines denote the median, boxes indicate the interquartile range (IQR), whiskers indicate the extended range of 1.5× IQR
.

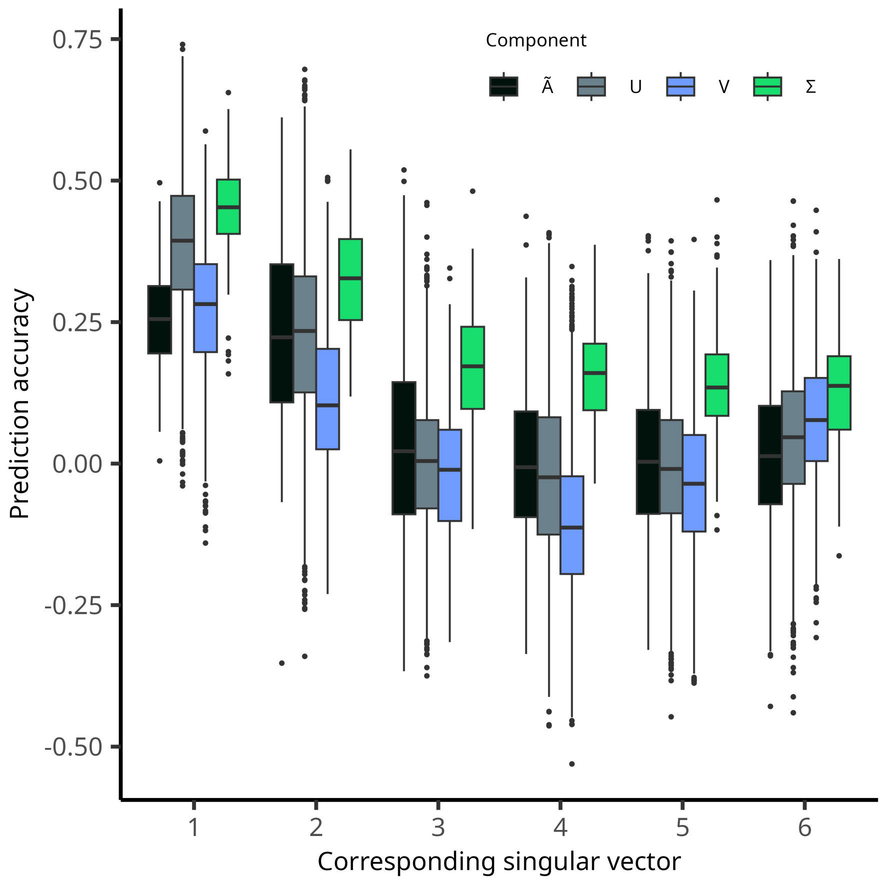


**Supplementary Figure 12**. **Predictability of elements of $\tilde{\mathbf{A}}$ as well as singular values and singular vector elements of $\mathbf{X}_1$ on a data set of an *A. thaliana* diversity panel.** The prediction accuracy for elements of $\tilde{\mathbf{A}}$ and the elements of the singular values and singular vectors of $\mathbf{X}_1$ using RR-BLUP drops to near 0 after the first two singular values, with the exception of the values of sigma, which remain above 0.1 up to six singular values. Horizontal lines denote the median, boxes indicate the interquartile range (IQR), whiskers indicate the extended range of 1.5× IQR.

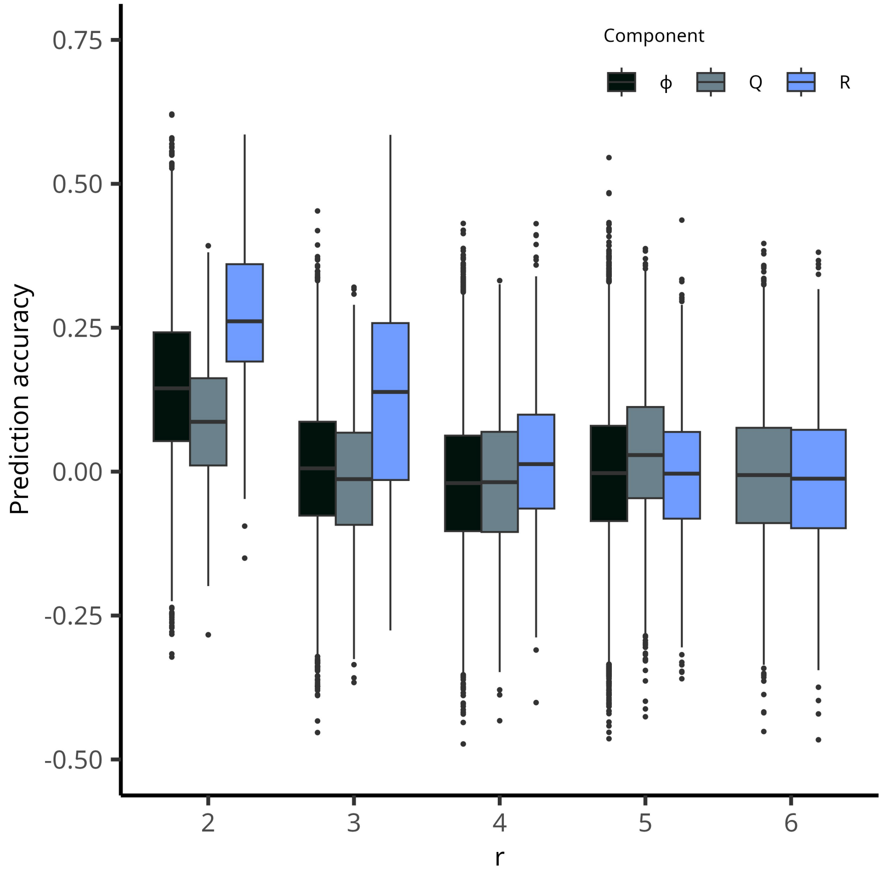


**Supplementary Figure 13**. **Predictability of the elements of Schur decomposition components and projected DMD modes on a data set of an *A. thaliana* diversity panel.** The prediction accuracy of the elements of Schur decomposition components of $\tilde{\mathbf{A}}$ ($\mathbf{Q}$ and $\mathbf{R}$) and the elements of the projected DMD modes ($\mathbf{\Phi}$) decreased to nearly zero for $r > 3$, where $r$ represents the number of singular vectors included in the truncated model obtained from algorithm 2. The accuracy for $\mathbf{Q}$ and $\mathbf{R}$ elements dropped to near 0 above $r > 2$. Horizontal lines denote the median, boxes indicate the interquartile range (IQR), whiskers indicate the extended range of 1.5× IQR.

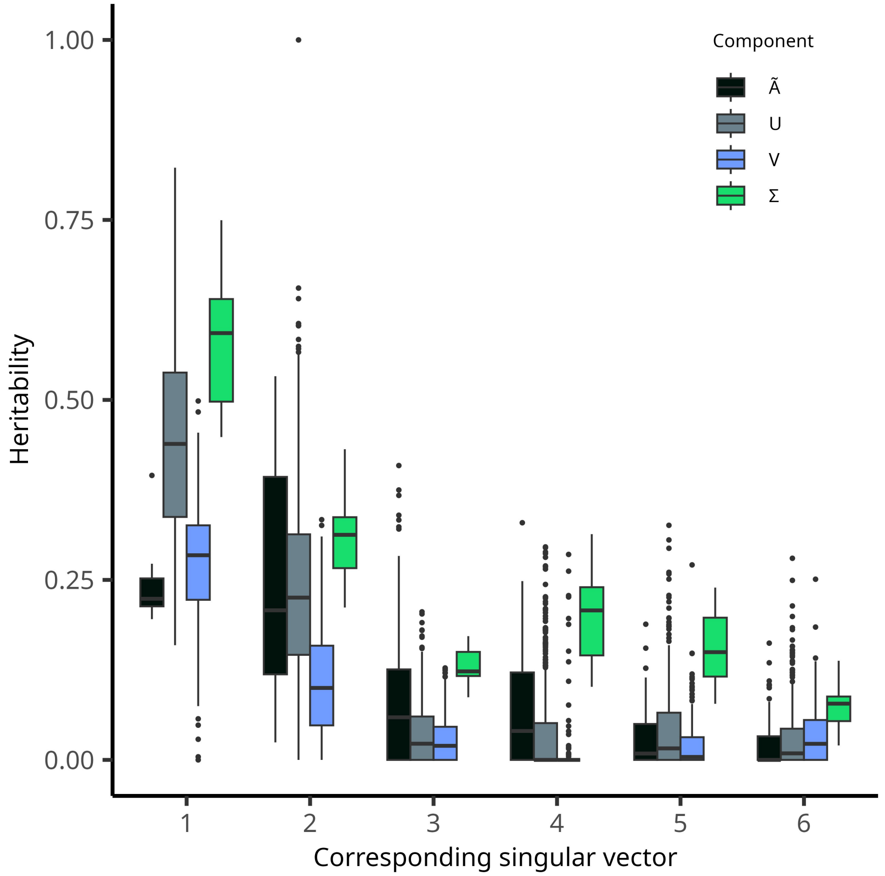


**Supplementary Figure 14**. **Heritability of elements of $\tilde{\mathbf{A}}$ as well as the singular values and singular vector elements of $\mathbf{X}_1$ in a validation configuration on a data set of an *A. thaliana* diversity panel.** The heritability of vector elements decreases to nearly zero after the first two singular values (SVs) in 10 iterations of training sets. This provides a mechanism for selection of *r*, denoting the number of included singular vectors in the truncated model obtained from Algorithm 2. Horizontal lines denote the median, boxes indicate the interquartile range (IQR), whiskers indicate the extended range of 1.5× IQR.

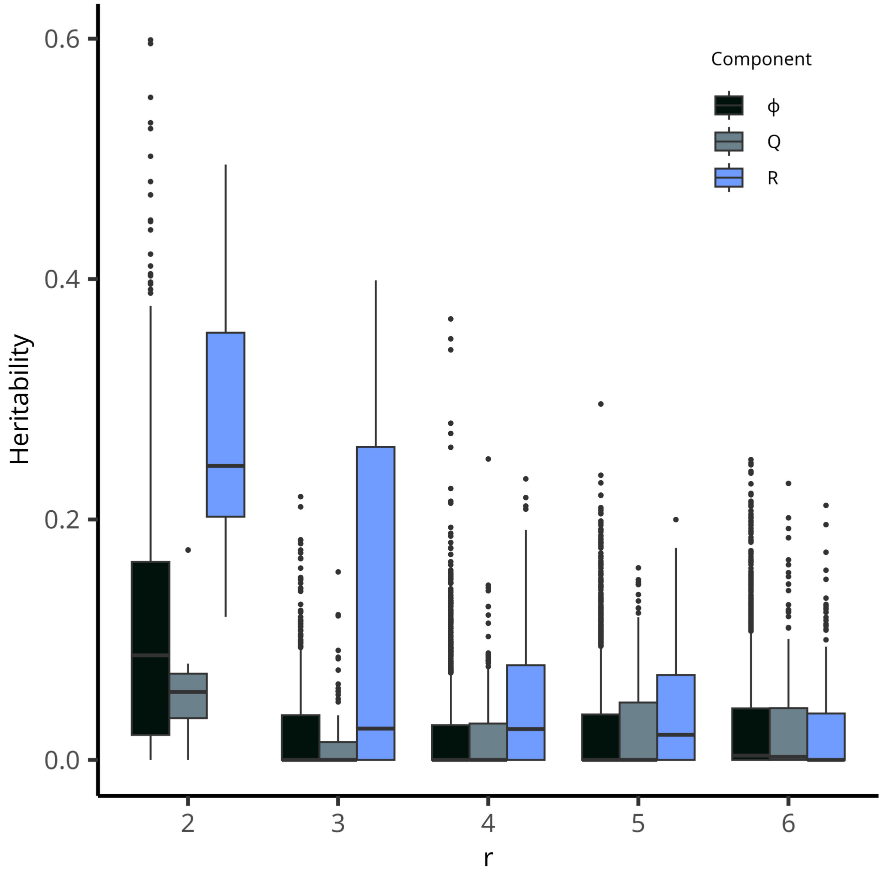


**Supplementary Figure 15**. **Heritability of the elements of Schur decomposition components and projected DMD modes in a validation configuration on a data set of an *A. thaliana* diversity panel.** The heritability of the elements of Schur decomposition components of $\tilde{\mathbf{A}}$ **(Q** and **R)** and the elements of the projected DMD modes ($\mathbf{\Phi}$) decreased to nearly zero for $r > 2$, where $r$ represents the number of singular vectors included in the truncated model obtained from Algorithm 2. Horizontal lines denote the median, boxes indicate the interquartile range (IQR), whiskers indicate the extended range of 1.5× IQR.

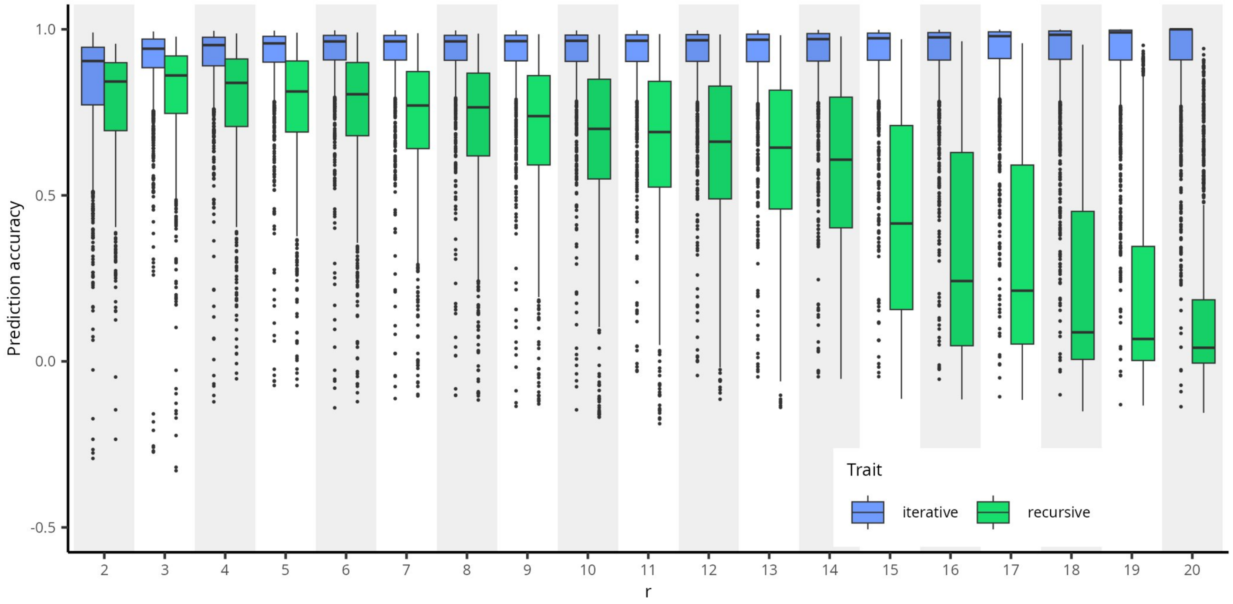


**Supplementary Figure 16**. **Performance of DMD Algorithm 2 with different number of singular vectors retained on the maize data set.** Larger number of retained singular vectors, $r$, results in a higher prediction accuracy in recreating training data with the iterative version (blue). However, it leads to lower accuracies when using the recursive version (green), due to the higher degree of over-fitting to the training data. The latter is revealed when predicting the time points immediately after the two-day gaps (i.e. after $t = [5, 10, 15, 20]$). Each box represents the mean accuracy across all 50 traits and all 25 time points for a given value of $r$. Horizontal lines denote the median, boxes indicate the interquartile range (IQR), whiskers indicate the extended range of 1.5× IQR.
